# Multifunctional combined drug-loaded nanofibrous dressings with anti-inflammatory, antioxidant stress and microenvironment improvement for diabetic wounds†

**DOI:** 10.1039/d4ra04860a

**Published:** 2024-09-18

**Authors:** Yuqing Ju, Yuxuan Luo, Ruimeng Li, Wei Zhang, Yan Ge, Jiapeng Tang

**Affiliations:** a Institute of Special Environmental Medicine, Nantong University Nantong 226019 PR China jptang@ntu.edu.cn; b Co-innovation Center of Neuroregeneration, Nantong University Nantong 226001 PR China; c School of Textile and Clothing, Nantong University Nantong 226019 PR China; d National & Local Joint Engineering Research Center of Technical Fiber Composites for Safety and Protection, Nantong University Nantong 226019 PR China

## Abstract

The treatment of diabetic wounds remains a formidable clinical challenge worldwide. Because of persistent inflammatory reaction, excessive oxidative stress, cell dysfunction, poor blood microcirculation and other microvascular complications, diabetic wounds often fall into inflammatory circulation and are difficult to heal, making patients confront the risk of amputation. In this study, silver complex nanoparticles with Resina Draconis extract and *Rhodiola rosea* L. extract were loaded *in situ* onto thermoplastic polyurethane nanofibers to develop a multifunctional electrospun nanofiber wound dressing with excellent mechanical properties, superior water absorption and breathability, good coagulation promoting activity, strong antibacterial performance and antioxidant properties. This wound dressing could effectively enhance the migration and proliferation of fibroblasts, reduce the increased thickness of regenerated epidermis caused by diabetes, and the high expression and high lipid peroxidation levels of IL-1 β, IL-6, TNF α, iNOS and MMP-9, and raise the low expression of VEGF, which shows great potential to accelerate the wound healing of diabetic mouse models. The wound healing rate reached about 87.92%, close to the non-diabetic group. Our findings suggest a breakthrough in diabetic wound care, offering a viable solution to a long-standing medical shackle.

## Abstract 2

The treatment of diabetic wounds remains a formidable clinical challenge worldwide.

## Graphical abstract

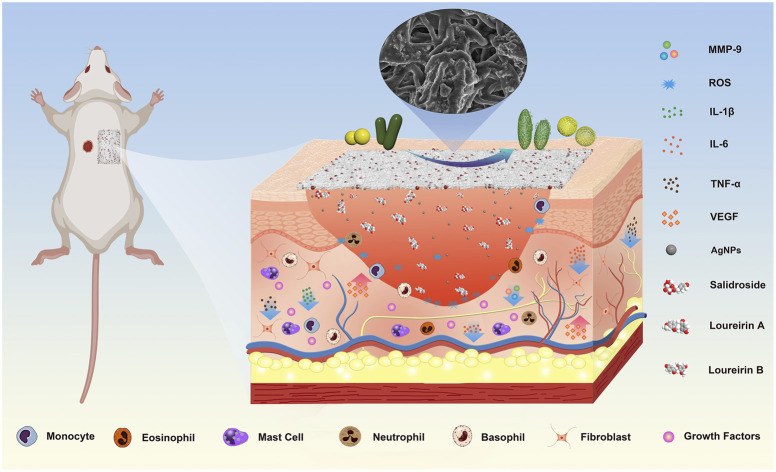

## 1 Introduction

Diabetes is a metabolic disease characterized by hyperglycemia, which is caused by the secretion defect or impaired biological function of insulin, or both.^1^ There had been 537 million (10.5%) diabetic patients aged 20–79 in the world by 2021, which is expected to increase to 783 million (12.2%) by 2045.^2^ Diabetes has many common complications, including cardiovascular disease, stroke, peripheral vascular disease, hypertension, retinopathy, kidney disease, diabetic ketoacidosis, peripheral neuropathy and foot ulcer.^1,3–5^ Among them, foot ulcer which is difficult to heal often leads to lower limb amputation, seriously affecting the quality of life for patients.^6^ So far, diabetic wounds can only be treated by the conventional wound treatment and few widely successful clinical treatments are available for such wounds. The key issue is the lack of suitable wound dressings during the treatment process.

After hemostasis, inflammation is one of the most important stages in wound healing.^7^ During the inflammatory process, immune cells are activated and release various bioactive substances, including cytokines and chemokines.^8^ These substances trigger more immune cells and repair cells to enter the wound area in order to clear pathogens and necrotic tissue, providing necessary conditions for the subsequent repair.^9^ However, stagnation in the inflammatory phase is one of the important reasons why diabetic wounds are difficult to heal.^10,11^ The long-term wound inflammation causes the dysfunction of immune cells,^12^ weakened vascular perfusion,^13^ thickened extracellular basement membrane and collagen synthesis dysfunction, thus hindering wound healing.^9,14^ Meanwhile, the hyperglycemic microenvironment on the diabetic wound surface is more prone to microbial infection than other types of wounds, which will also significantly delay the wound healing of diabetic patients.^14,15^ Based on modern pharmacological research, it has been found that Resina Draconis contains a variety of active ingredients, such as flavonoids, triterpenes, glycosides, stilbenes, organic acids, phenols and esters.^16^ The two most important flavonoids of them are loureirin A and loureirin B.^17^ Resina Draconis shows pharmacological effects such as anti-inflammatory, analgesic, antibacterial, and hemostatic properties and accelerating wound-healing. Traumatic bleeding, abscess, refractory ulcer, hemorrhoids and pressure sores can all be prominently relieved and cured through the local application of Resina Draconis.^18^

In the process of wound healing, oxidative stress will occur accompanied by the production of reactive oxygen species (ROS). A small amount of ROS can stimulate the release of some cytokines and promote the migration and proliferation of fibroblasts.^19^ However, the oxidative stress response at the wound site can be exacerbated in the case of diabetes, leading to the excessive production of ROS.^20,21^ The presence of these excessive ROS may damage DNA, RNA and proteins, causing lipid peroxidation (LPO).^22–24^ LPO can cause the poor prognosis of healing in diabetic wounds. Firstly, LPO can damage blood vessels around the wound,^25^ leading to poor blood circulation and affecting the oxygen and nutrition supply to the wound. Secondly, LPO can inhibit fibroblastic proliferation and collagen synthesis in the wound, delaying the wound healing.^26^ In addition, LPO can enhance inflammatory reactions,^27^ making wounds more susceptible to infection and further prolonging healing time. Salidroside, the main active ingredient of *Rhodiola rosea* L., can inhibit LPO by clearing superoxide anions (·O-2), hydroxyl radicals (·OH) and lipid-derived free radicals, and enhance the activities of superoxide dismutase (SOD), glutathione peroxidase (GSH Px) and catalase (CAT) to exert its antioxidant effect.^28–32^ In terms of anti-inflammatory effects, salidroside produces anti-inflammatory effects by affecting the distribution of lymphocytes and inhibiting the activation and migration of inflammatory cells and their infiltration into tissues and organs, which markedly decreases the levels of IL-1β, IL-6 and TNF-α and rebalanced inflammatory chemokines, thereby reducing inflammatory response.^28,33–35^

Diabetic patients are vulnerable to bacterial invasion due to the decline of immune system function and abnormal skin tissue structure. When bacteria enter the wounds, they further trigger an inflammatory response, leading to symptoms such as swelling, redness, and pain in the surrounding tissue of the wound, and prolonging its healing time.^15^ The development of antibiotics has further helped to combat many pathogens. However, bacteria gradually adapted to them and gained resistance owing to the abuse of antibiotics, resulting in a large number of drug-resistant strains. The therapeutic effect of antibiotics is getting worse and the side effects are becoming more prominent, making it difficult to control wound infections.^36^ The development of novel metal-based molecular antibiotics is an effective strategy to combat the risk of microbial diseases. Metal-based molecular antibiotics are a type of transition metal complex. The previous studies showed that complexes such as cobalt, ruthenium, manganese and vanadium had various physiological activities including antibacterial, antioxidant, anticancer and hypoglycemic effects.^37–41^ With the increase of chelation, the charge was transferred from the metal to the ligand, enhancing physiological activities in antibacterial and antioxidant properties of transition metal complexes. In addition, silver nanoparticles have become the most promising inorganic antibacterial agents in virtue of their excellent antibacterial property, non-cytotoxicity and no drug-resistance, and have been widely studied and applied in wound dressings.^42,43^ The use of novel silver complex nanoparticles (AgNPs) for antibacterial and antioxidant applications has not been reported yet.

Thermoplastic polyurethane (TPU) is a polymer with high strength, toughness, wear resistance and oil resistance, which has been widely used in industries such as textiles, food and national defense.^44^ Thanks to its high biodegradability, mechanical properties and biocompatibility, TPU has also been applied extensively in the biomedical field, such as biomimetic fingers, vascular grafts and heart valves.^45–48^ Considering its pathogen barrier and oxygen permeability, it is also used as an alternative wound dressing. The TPU electrospun nanofiber membrane possesses the characteristics of large specific surface area, high porosity, small pore size and adjustable composition.^49,50^ Its porous structure is similar to that of the natural extracellular matrix (ECM), which is conducive to gas exchange and exudate absorption at the wound site, thereby enhancing the regeneration of skin tissue in the wound area.^51^ The nanofiber membrane can also load therapeutic drugs or active ingredients to achieve controlled drug release.^52,53^ This provides a huge application prospect for the treatment of diabetic wounds.^54^

An ideal diabetic wound dressing needs to meet the following requirements: inducing an acute immune reaction, decreasing chronic inflammation, interfering ROS generation, inhibiting or down-regulating matrix metalloproteinase-9 (MMP-9), reducing the damage to growth factors, ECM and stromal cells, resulting in substituting the damaged ECM, providing direct interaction sites for fibroblasts and achieving fibroblast activation and the secretion of growth factors so as to develop unhealed wounds towards healing.^55^ In this work, we prepared TPU nanofiber membranes loaded with AgNPs coordinated by the extracts of Chinese medicinal materials including Resina Draconis and *Rhodiola rosea* L. to promote diabetic wound healing by alleviating inflammation in diabetic mice, preventing bacterial infection, decreasing the oxidative stress level and LPO level, and accelerating neovascularization.

## 2 Materials and methods

### 2.1 Materials

Chemicals and reagents used in the study were sourced as follows: Resina Draconis and *Rhodiola rosea* L. were obtained from Nantong Local Pharmacy, while TPU was purchased from Baoding Bangtai Polymer New Material Co., Ltd (Hebei, China). Silver nitrate was acquired from Sinopharm Chemical Reagent Co., Ltd (Shanghai, China), and *N*,*N*-dimethylformamide (DMF) and tetrahydrofuran (THF) were procured from Shanghai Lingfeng Chemicals Co., Ltd (Shanghai, China). All reagents were used without further purification. Streptozocin (STZ) was purchased from Shanghai Macklin Biochemical Technology Co., Ltd (Shanghai, China).

Cell lines, including mouse embryonic fibroblast cells (NIH3T3) and mouse epithelioid fibroblast cells (L929), were sourced from Procell Life Science & Technology Co., Ltd (Wuhan, China). The medium required for cell culture was Dulbecco's modified Eagle medium (DMEM, Gibco, US). The study also involved microorganisms: *Escherichia coli* (*E. coli*, ATCC 8739), *Staphylococcus aureus* (*S. aureus*, ATCC 6538) and *Candida albicans* (*C. albicans*, ATCC 10231) were obtained from Beijing Baioubowei Biotechnology Co., Ltd (Beijing, China), while *Pseudomonas aeruginosa* (*P. aeruginosa*, ATCC 9027) was acquired from Hangzhou Fenghai Biological Technology Co., Ltd (Hangzhou, China).

Male ICR mice with SPF (Specific Pathogen-Free) status, aged 8 weeks and weighed between 20 and 35 g, were sourced from Animal Center of Nantong University and used in accordance with the protocols approved by Animal Care and Use Committee of Nantong University. All animal procedures were performed in accordance with the Guidelines for Care and Use of Laboratory Animals of Nantong University and approved by the Animal Care and Use Committee of Nantong University and the Jiangsu Province Animal Care Ethics Committee (Approval ID: S20220219-010).

### 2.2 Fabrication of TPU nanofiber membranes

The components of Resina Draconis and *Rhodiola rosea* L. were extracted by the Soxhlet extraction method. Briefly, ethyl acetate and ethanol were used as extraction solvents to extract traditional Chinese medicinal materials containing Resina Draconis and *Rhodiola rosea* L., respectively. The solvents were then removed by rotary evaporation to obtain Resina Draconis extract (Rd) and *Rhodiola rosea* L. extract (Rc). The extraction rates of Rd and Rc were 42% and 35%.

1.8 g TPU was dissolved in 10 ml of a reagent mixture of DMF and THF (1 : 1 v/v) to obtain an 18% (w/v) polymer solution and stirred with a magnetic stirrer to form a uniform spinning solution, whose viscosity was 10.20 ± 0.35 Pa s. Then, the resultant solution was electrospun at a distance of 15 cm between the needle tip and drum collector, a voltage of 15 kV and a flow rate of 0.5 ml h^−1^. A layer of silicone oil paper was wrapped on the drum to collect electrospun nanofibers and the rotating speed of the drum collector was 500 rpm. The prepared nanofibers were labelled as TPU@None. Additionally, 50 mg Rd and Rc were separately or simultaneously added into the spinning solution above, and the fabricated resulting nanofiber membranes were labelled as TPU@Rd, TPU@Rc and TPU@Rd_Rc.

The electrospun TPU@Rd_Rc was sequentially immersed in 0.005 mol L^−1^ AgNO_3_ solution and 0.1 mol L^−1^ NaOH solution, and then rinsed with deionized water to remove the residual NaOH. Finally, the nanofiber membrane was obtained after drying and labelled as TPU@nAg#Rd_Rc.

The thicknesses of TPU@None, TPU@Rd, TPU@Rc, TPU@Rd_Rc and TPU@nAg#Rd_Rc were 0.27 ± 0.02 mm, 0.29 ± 0.04 mm, 0.28 ± 0.03 mm, 0.30 ± 0.05 mm and 0.25 ± 0.03 mm, respectively, which were stored in a self-zip plastic bag and placed in an environment of 4 °C.

### 2.3 Characterization

#### 2.3.1 Scanning electron microscopy

TPU nanofiber membranes cut into approximately 0.5 cm × 0.5 cm in size were fixed on the sample stage. The morphology and structure of TPU nanofibers were observed through scanning electron microscopy (SEM; GeminiSEM 300, Carl Zeiss, Germany) after vacuum platinum plating treatment (EM ACE600; Leica, Germany) and energy spectrum analysis was used to confirm the elemental composition and distribution of nanofibers. The diameter of nanofibers was measured using the image analysis software Image J (version 1.5.2). 50 readings were randomly taken and measured for each sample and the means were calculated.

#### 2.3.2 Fourier transform infrared spectroscopy

The chemical structure of nanofiber samples was assayed using Fourier transform infrared (FTIR) spectroscopy (TENSOR 27, Bruker, Germany). All spectra were collected by transmission mode in the wavelength range of 4000–400 cm^−1^ in a dry atmosphere at room temperature.

#### 2.3.3 Thermogravimetry and differential scanning calorimetry

The combination of thermogravimetry (TGA) and differential scanning calorimetry (DSC) of TPU nanofiber samples were performed using a synchronous thermal analyzer (STA 449 F5, NETZSCH, Germany). It was operated in static mode under atmospheric air at a heating rate of 10 °C min^−1^ in a temperature range of 30–600 °C.

#### 2.3.4 X-ray diffraction

X-ray diffraction (XRD) was used to analyze the crystallization characteristics of the prepared nanofiber samples using a nickel-filtered Cu-Ka radiation-assisted XRD system (Ultima IV, Rigaku, Japan). The scanning speed was maintained at 5° min^−1^ and the 2*θ* range was located from 5° to 80°.

### 2.4 Physicochemical properties

#### 2.4.1 Mechanical test

The mechanical properties of TPU nanofiber membranes were tested using a smart electronic tensile testing machine (BAB-5MT, Shanghai Xiangjie, China). The samples were cut into strips measuring 100 mm × 10 mm at a stretching speed of 100 mm min^−1^. Each sample was tested three times to calculate the average value and standard deviation.

#### 2.4.2 Water contact angle

TPU nanofiber membranes were cut into 10 cm × 1 cm strips. A contact angle goniometer (JC2000C2, POWEREACH, China) was used to deposit a 5 microliter droplet of deionized water on each sample's surface, capturing a side-view image to determine the contact angle. This measurement was repeated three times for each sample. The average contact angle was recorded and its standard deviation was calculated.

#### 2.4.3 Swelling ratio

After drying to a constant weight, TPU nanofiber membranes were weighed (*W*_d_) and then immersed in a 0.01 M phosphate buffered saline (PBS) solution at pH 7.4. The nanofiber samples were taken out and weighed (*W*_s_) at regular intervals. Swelling ratio (SR) of nanofibers was calculated using eqn (1).1
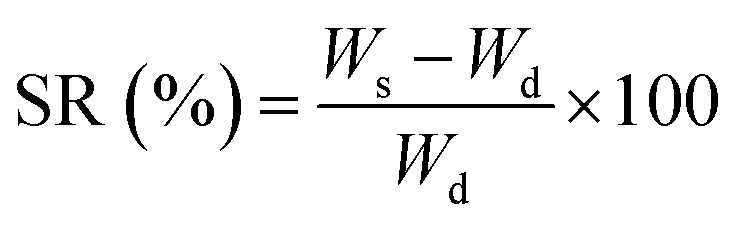


#### 2.4.4 Water vapor transmission rate

0 ml water was added into a 30 ml glass bottle with a top inner diameter of 2.4 cm (*d*). The different TPU nanofiber membranes cut into small pieces covered the bottle mouth and the whole weight (*W*_i_) was weighed. Then, the bottle was put into a 37 °C constant temperature incubator and the whole weight (*W*_f_) was re-weighed after 24 h. The calculation formula for water vapor transmission rate (WVTR) is shown in eqn (2).2
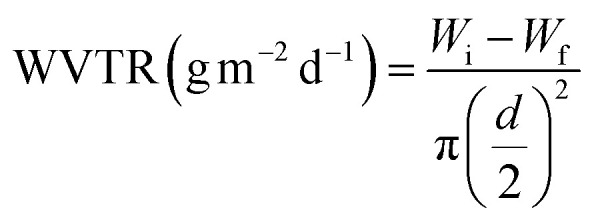


#### 2.4.5 Release behaviour

TPU@Rc and TPU@Rd_Rc was respectively cut and weighted 0.5 g, then added 20 ml serum to place in a shaker at 37 °C with a shaking speed of 200 rpm. The samples were taken at regular intervals and the contents of salidroside, loureirin A and loureirin B were detected by high performance liquid chromatography (HPLC, Alliancee2695, Waters, USA).

### 2.5 Tube test for biological function

#### 2.5.1 Antibacterial activity

Four strains, Gram-positive bacteria (*S. aureus* ATCC 6538), Gram-negative bacteria (*E. coli* ATCC 8739 and *P. aeruginosa* ATCC 9027) and fungi (*C. albicans* ATCC 10231) were chosen for antimicrobial test of TPU nanofiber membranes by the oscillation method. Bacteria were cultured in Luria–Bertani medium (LB) and fungi were cultured in yeast extract peptone dextrose medium (YPD). TPU nanofiber membranes cut into a wafer of about 3 cm diameter were placed in tubes with 5.0 ml LB and YPD broth, followed by sterilization. The bacterial and fungal broths were incubated until their optical density (OD) 600 nm reached 0.5. Then, the broth was added to each tube containing the samples. The tubes were then incubated for 12 h at 37 °C in a shaking incubator. Finally, 20 μl liquid in the tubes was coated on LB agar and YPD agar and incubated overnight at 37 °C. The culture was photographed and the bacterial colonies were counted for calculating the antibacterial rate. The antibacterial rate of membranes was calculated by the following eqn (3):3
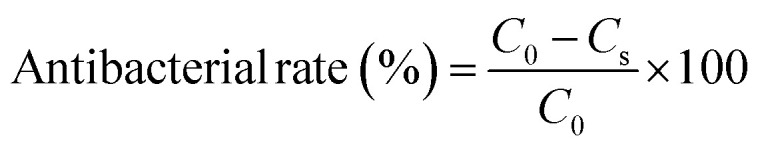
where *C*_0_ and *C*_s_ are the bacterial colonies in agar culture medium without and with adding TPU nanofiber membranes, respectively.

#### 2.5.2 Antioxidant activity

20 mg TPU nanofiber membranes were put into 3 ml DPPH solution and reacted at room temperature in the dark for 30 min. Then, the absorbance value of resultant solutions was measured by using a UV spectrophotometer (UV-2450 spectrophotometry, SHIMADZU, Japan) at a wavelength of 517 nm using ethanol as a blank. DPPH radical clearance rate was calculated by eqn (4):4
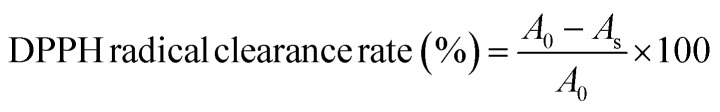
where *A*_0_ is the absorbance value of the blank group and *A*_s_ is the absorbance value of the sample group.

#### 2.5.3 Blood compatibility and coagulation performance

In order to investigate whether the prepared TPU nanofiber membranes had blood compatibility, the hemolysis experiment was used to evaluate whether the nanofibers induced hemolytic behaviour. Red blood cell suspension (RBCS) was obtained by centrifugation of SD rat anticoagulant whole blood and PBS washing. Each sample was cut into 1 cm × 1 cm in size and added 300 μl RBCS and 1 ml PBS. All samples were incubated at 37 °C for 3 h and then centrifuged to collect the supernatant. OD value of the obtained supernatant was measured at a wavelength of 542 nm (OD_t_). The system without the sample was the negative control (OD_nc_) and that using deionized water instead of PBS was the positive control (OD_pc_). The calculation formula of hemolysis ratio is shown in eqn (5).5
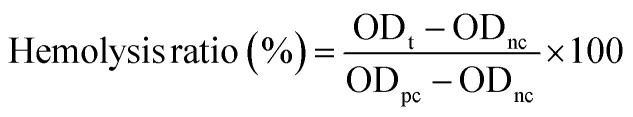


Blood coagulation time and blood coagulation index (BCI) were used to evaluate their coagulation performance to investigate whether the nanofibers had the ability to promote coagulation. The nanofiber samples cut into 1 cm × 0.5 cm were placed in centrifuge tubes and put in a 37 °C constant temperature incubator for 5 min. 100 μl anticoagulant whole blood and 20 μl 0.2 M CaCl_2_ solution were added to each well and kept in a 37 °C constant temperature incubator. The blood coagulation time was recorded.

Additionally, the nanofiber samples cut into 1 cm × 1 cm were put into the culture dish and incubated at 37 °C for 5 min. 100 μl anticoagulant whole blood and 20 μl 0.2 M CaCl_2_ solution were added to each sample and then incubated at 37 °C for 10 min. 5 ml deionized water was then added and shaken for 5 min. Finally, the liquid was collected after removing the samples. The absorbance of the resultant liquid was measured by using an ultraviolet spectrophotometer (UV-2450 spectrophotometry, SHIMADZU, Japan) at 542 nm. BCI was calculated by the following eqn (6):6
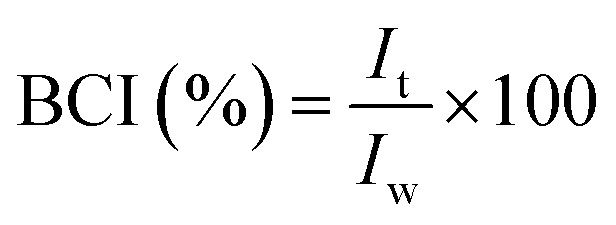
where *I*_t_ is the OD value of the liquid with the treatment of samples and *I*_w_ is OD value of liquid without adding samples.

#### 2.5.4 Cell viability and migration

L929 cells and NIH3T3 cells were cultured in DMEM supplemented with 10% fetal bovine serum (FBS) and 1% penicillin–streptomycin in a humidified incubator containing 5% CO_2_ at 37 °C. To assess the toxicity of TPU nanofiber membranes, cells were initially inoculated into 96-well plates at a concentration of 4000 cells per well. After cell adhesion, the culture medium in the wells was replaced by the medium containing TPU nanofiber leachate. Following a 24 hours incubation, the medium was discarded and the wells were rinsed with PBS. Each well then received 100 μl of serum-free medium with 3-(4,5-dimethylthiazol-2-yl)-2,5-diphenyltetrazolium bromide (MTT, 0.5 mg ml^−1^) and incubated for 4 h at 37 °C. 15% SDS was added to dissolve the formazan crystals overnight post incubation. OD at 570 nm was measured for each well using a microplate reader (Synergy2, BioTek Instruments, USA). Each sample was tested three times. Both the average value and standard deviation were calculated.

In addition, 8 × 10^4^ cells were inoculated into 6-well plates. When the fusion degree of cells in 6-well plates reached 90%, straight lines were drawn at the bottom of wells with pipette tips of 10 μl. The culture medium containing 1% FBS and TPU nanofiber leachate was replaced for starvation cultivation. The images of each scratch were recorded under a microscope (DMIL LED, Leica, Germany) after 0 h and 24 h, and the average scratch area was calculated using Image J (version 1.5.2) for statistics.

#### 2.5.5 Anti-inflammatory and anti-LPO properties

NIH3T3 cells were cultivated in DMEM supplemented with 10% FBS and 1% penicillin–streptomycin until the cells fully overspread the culture dish. Then, the cells were digested by trypsin and inoculated into 12-well plates at a concentration of 4.0 × 10^4^ cells per ml. After the cells adhered to the wall, the culture medium was removed and washed twice with PBS, and then 1 ml of 1 μg ml^−1^ lipopolysaccharide (LPS) solution was added into the well for treatment for 7 h. Next, the LPS solution was removed and 1 ml of culture medium containing TPU nanofiber leachate was added to each well and cultured for another 24 h. Subsequently, cellular proteins and RNA were extracted for western blot analysis, MDA assay and qPCR experiments.

### 2.6 Animal experiment

#### 2.6.1 Wound model in diabetic mice and evaluation of *in vivo* wound healing effect

Eight-week-old male ICR mice were fasted the night before the experiment and received a single intraperitoneal injection of streptozocin (STZ) (50 mg kg^−1^) for four consecutive days. STZ solution protected from light was kept in an ice bath, and the injection process was completed within 30 min. The mice had free access to food and water post-injection. Diabetes was confirmed seven days later through two consecutive blood glucose readings of ≥16.8 mmol l^−1^, along with classic diabetic symptoms. A wound model was established after maintaining elevated blood glucose for seven days. Healthy mice were used as a control group and anesthetized with 2.5% Avertin solution. A 5 mm diameter full-thickness skin excision was made on their backs using a skin punch tool and treated with standard medical gauze. Diabetic mice successfully modelled were randomly divided into six groups and underwent a similar wound model. They were treated with standard medical gauze, TPU@None, TPU@Rd, TPU@Rc, TPU@Rd_Rc or TPU@nAg#Rd_Rc. Wound healing was photographed and recorded on days 0, 3, 5, 7, 10 and 14, respectively. The calculation formula of wound healing rate (WR) is shown in eqn (7):7
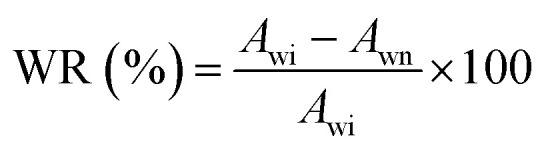
where *A*_wi_ is the initial wound area, cm^2^, and *A*_wn_ is the wound area after healing for 3, 5, 7, 10, and 14 days, cm^2^.

#### 2.6.2 Tissue staining

After the different treatments of diabetic mice for 7 and 14 days, the mice were killed and the skin tissue of the wound was removed to be fixed overnight with 4% paraformaldehyde, dehydrated with ethanol of gradient concentration, cleared in xylene and embedded in paraffin for sectioning at 6 μm thickness. The obtained slices were subjected to H&E staining, Masson staining and immunohistochemical staining with inducible nitric oxide synthase (iNOS) and MMP-9 using healthy mice as controls.

#### 2.6.3 LPO levels

The healthy and diabetic mice were killed after different treatments for 7 and 14 days. Their removed wound skin tissue was milled. The content of MDA was detected with MDA kit.

#### 2.6.4 Inflammatory factors and VEGF expression levels

After different treatments for 7 days in diabetic mice, the mice were euthanized and skin tissue from the wound site was collected. The tissue was then ground to extract RNA for qPCR experiments in order to assess the changes in expression levels of *Il6*, *Il1b* and *Tnfa*. Additionally, proteins were extracted for western blot analysis to monitor the changes in VEGF expression.

## 3 Results and discussion

### 3.1 Physicochemical characterization

#### 3.1.1 Morphological structure of nanofibers

**Fig. 1:**
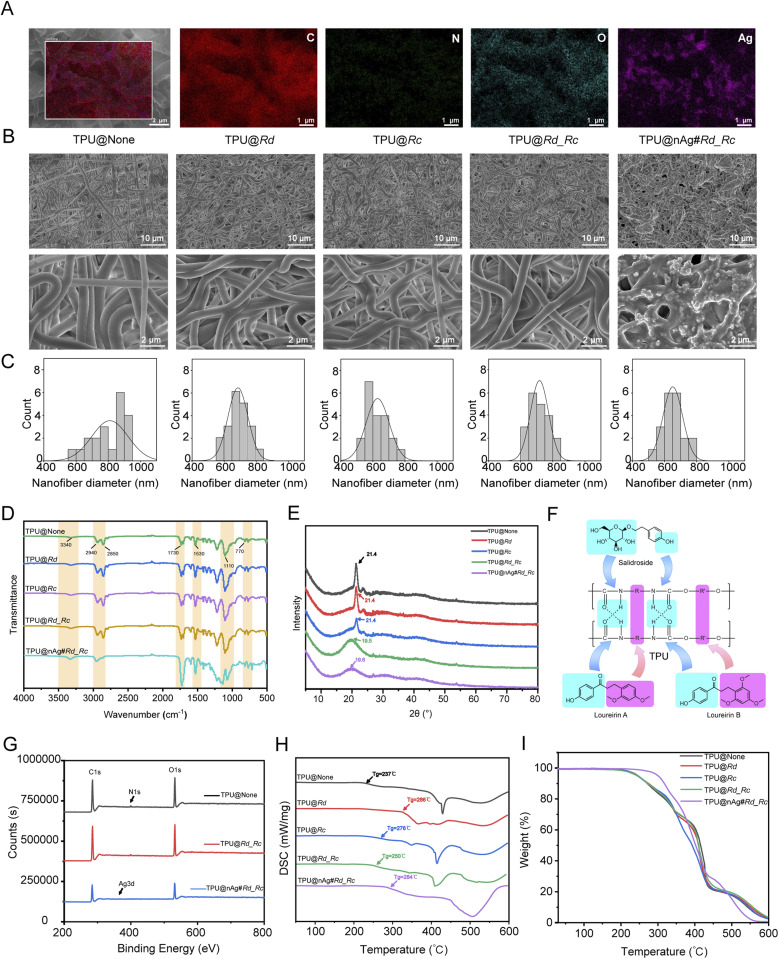
(A) EDS elemental analysis of TPU@nAg#Rd_Rc; (B) SEM images of different TPU nanofibers; (C) statistical diameters of different TPU nanofibers; (D) infrared spectra of various TPU nanofibers; (E) XRD analysis of TPU nanofibers; (F) schematic diagram of the intermolecular interactions between TPU, salidroside, loureirin A and loureirin B; (G) XPS analysis of TPU nanofibers; (H) DSC analysis of TPU nanofibers; (I) TGA analysis of TPU nanofibers.

The morphology, fiber diameter and uniformity of TPU nanofibers were evaluated using SEM. According to EDS elemental analysis (Fig. 1A), there existed not only the distribution of C, N, and O elements on TPU@nAg#Rd_Rc, and silver elements also clustered along the fibers, indicating the successful loading of AgNPs. Fig. 1B indicated that five different TPU nanofiber membranes including TPU@None, TPU@Rd, TPU@Rc, TPU@Rd_Rc and TPU@nAg#Rd_Rc exhibited a uniform and smooth morphology with a good structure, without beading or adhesion, and their diameters all reached the nanoscale. Fig. 1C displayed that the blank TPU fiber diameter was the largest, about 810 ± 110 nm, and the fiber distribution was relatively dispersed, while the diameter of TPU fibers loaded with Rd or Rc was significantly decreased compared to TPU@None (Fig. S1†) and the dispersion degree became lower, indicating that the loading of two drug extracts could effectively reduce the diameter of TPU nanofibers and make the fiber distribution more uniform. The diameter of AgNPs was 359 ± 67 nm (Fig. S2†) and that of TPU@nAg#Rd_Rc loaded with AgNPs was 651 ± 59 nm, which was significantly smaller than that of TPU@Rd_Rc (Fig. S1†). The reason might be due to the etching effect of the sequential treatment with silver nitrate solution and sodium hydroxide solution on TPU nanofibers, resulting in a further decrease in the fiber diameter. As the fiber diameter decreased, the specific surface area and porosity increased, which could be more conducive to simulating the natural ECM structure.^56^ This porous network structure could profit gas exchange and exudate absorption at the wound site, thereby promoting the regeneration of skin tissue in the wound area to effectively accelerate wound healing.^57^ The above results declared the successful preparation of TPU nanofiber membranes loaded with various drugs.

#### 3.1.2 Chemical structure of nanofibers

FTIR was used to determine the functional groups in Rd, Rc and TPU nanofibers to understand the interactions among the components of TPU nanofibers. In the infrared spectrum of Rd (Fig. S3†), the broad and strong absorption peak at 3340 cm^−1^ was attributed to O–H in the hydroxyl group. The anti-symmetric stretching vibration absorption peak of C–H in methylene occurred at 2930 cm^−1^ and the bending vibration absorption peak of O–H appeared at 1600 cm^−1^. The stretching vibration absorption peaks of the benzene skeleton occurred at 1510 cm^−1^ and 1450 cm^−1^. The peaks at 1160 cm^−1^ and 1031 cm^−1^ were ascribed to the C–O stretching vibration absorption peaks. These peaks above were consistent with the main components contained in Resina Draconis, including phenols, flavonoids and glycosides. As shown in the infrared spectrum of Rc (Fig. S3†), the C–H stretching vibration of benzene occurred at 3219 cm^−1^ and the peak at 2920 cm^−1^ was attributed to the saturated C–H stretching vibration. The vibrations of the benzene skeleton appeared at 1612 cm^−1^, 1510 cm^−1^ and 1430 cm^−1^, and the C–O stretching vibration of the phenolic hydroxyl group occurred at 1220 cm^−1^. The stretching vibration peak at 1037 cm^−1^ was attributed to C–O–C. As seen from Fig. 1D, the peak at 3340 cm^−1^ of TPU@None was generated by the stretching vibration of N–H, which coincided with the peak of the hydroxyl group. The peaks at 2940 cm^−1^ and 2850 cm^−1^ were ascribed to the stretching vibration of C–H (CH_2_). The strong absorption peak at 1730 cm^−1^ was the result of the overlapping absorption of C=O by esters and polyurethane. The peak at 1530 cm^−1^ was generated by the C=C vibration of the benzene skeleton, and the absorption peak at 1100 cm^−1^ was caused by the C–O vibration of fatty acid esters. The peak at 770 cm^−1^ was attributed to the C–H vibration of benzene.^49,58^ In the FTIR spectra of several drug-loaded TPU nanofibers, the main absorption peaks of TPU@Rd, TPU@Rc, TPU@Rd_Rc and TPU@nAg#Rd_Rc did not shift compared to TPU@None and no new peaks appeared, indicating that TPU was the main structural component in drug-loaded nanofibers, which was the prerequisite for their stable physicochemical properties.

The crystallization of TPU nanofiber membranes was analyzed using XRD. As shown in Fig. 1E, it presented a distinct diffraction peak at 21.4° in TPU@None, which corresponded to the crystallization peak of polyurethane hard segments and the characteristic peak of TPU.^59^ When loaded with Rd or Rc alone, the diffraction peak of TPU nanofibers did not change, illustrating that there was no significant change in the crystal region of hard segments in TPU. When two drugs were added simultaneously, the diffraction intensity of TPU nanofibers weakened. It could be inferred that the intermolecular interactions of hard segments including the original hydrogen bonds were disrupted when two drug extracts were simultaneously incorporated (Fig. 1F), causing the decrease in crystallinity. TPU nanofiber membranes became more flexible and satisfied the requirements of wound dressings. The qualitative analysis of elements was conducted using XPS. The peaks of C 1s, N 1s and O 1s at 285 eV, 400 eV and 532 eV could be observed on the energy spectrum of TPU@None (Fig. 1G). The peak of Ag 3d at 367 eV could be seen after TPU nanofibers were loaded with AgNPs, indicating the successful loading of silver. The calculation results of element content in the samples based on XPS measurement spectra are also shown in Table S1.†

#### 3.1.3 Thermodynamic performance

DSC was used to study the glass transition temperature (*T*_g_) and the situation of degradation, thermal transformation, fusion and crystallization of various TPU nanofiber membranes.^60^ The larger *T*_g_ implied the better amorphous stability of nanofibers. As shown in Fig. 1H, the amorphous stability of TPU@Rd, TPU@Rc, TPU@Rd_Rc and TPU@nAg#Rd_Rc was superior to that of TPU@None. The above results indicated that the addition of Rd, Rc and AgNPs could improve the amorphous stability of TPU nanofibers. Thermal stability of TPU nanofiber membranes was also evaluated using TGA and the process was divided into three stages according to the inflection points during the derivative variation course after the extrapolation of the TGA curve (Fig. S4†). Fig. 1I showed that each TPU nanofiber began to experience mass loss and the weight loss curve was relatively gentle in the first stage. The weight loss rates of TPU@None, TPU@Rd, TPU@Rc, TPU@Rd_Rc and TPU@nAg#Rd_Rc were 36.05%, 37.04%, 19.02%, 35.49% and 27.17%, respectively. In the second stage, the front section of the weight loss curve was relatively flat and the curve began to change suddenly at around 400 °C, indicating that TPU nanofibers started to degrade. In the third stage, the thermal decomposition of TPU nanofibers was basically completed and their quality gradually stabilized. The thermodynamic parameters of TPU nanofibers are shown in Table S2.†

### 3.2 Mechanical properties

The mechanical properties of nanofiber membranes were evaluated using tensile experiments. As shown in Fig. S5A,† TPU@None, TPU@Rd, TPU@Rc, TPU@Rd_Rc and TPU@nAg#Rd_Rc exhibited excellent mechanical properties. The Young's modulus of TPU@None was 29.64 ± 0.9 kPa, while the Young's modulus of TPU@Rd_Rc and TPU@nAg#Rd_Rc was significantly decreased to 19.72 ± 2.6 kPa and 16.97 ± 0.4 kPa compared to TPU@None (Fig. S5B†), declaring that the co-loading of Rd and Rc reduced the Young's modulus of TPU nanofibers. The main reason was that the large amount of hydroxyl groups and hydrophobic regions in Rd and Rc broke the hydrogen bonds and hydrophobic interaction in the hard segments of TPU, causing the decrease in crystallinity. Moreover, the components of Rc were mainly hydrophilic, while the components of Rd contained hydrophobic parts. Their combined effect was more effective in destroying the crystal regions (Fig. 1F). Fig. S5C† showed that the elongation at break of TPU@None reached over 180%, which was 189.8 ± 10%. The elongation at break of TPU@Rd_Rc and TPU@nAg#Rd_Rc was significantly higher than that of TPU@None, reaching 259.29 ± 16.74% and 257.53 ± 6.61%, respectively. In addition, there was no significant change in the elongation at break of TPU@nAg#Rd_Rc compared to TPU@Rd_Rc, illustrating that the loading of AgNPs had no effect on the elongation at break of TPU nanofibers. The above results could be mutually confirmed with the XRD results, that is, the Young's modulus of nanofibers significantly decreased and their elongation at break significantly increased after adding the two extracts compared to TPU@None, indicating that TPU nanofiber membranes exhibited greater flexibility after co-loading drugs and less stress on skin wounds.^61,62^ These results showed that the fabricated TPU nanofiber membranes had excellent mechanical properties and could meet the requirements of wound healing.

### 3.3 Hydrophilicity and breathability

In order to evaluate the hydrophilicity and breathability of TPU nanofiber membranes, contact angle measurement, swelling rate testing and WVTR measurement were conducted to determine whether the TPU nanofibers we prepared had the ability to absorb the exudates, maintain a moist environment at the wound site and improve the ability of wounds to uptake oxygen. As shown in Fig. S5D,† the contact angle of TPU@None was 135.1 ± 1.4°, exhibiting hydrophobicity. The contact angle of TPU@Rd decreased to 79.7 ± 2.1° by loading Rd and it changed from hydrophobicity to hydrophilicity. Similarly, the contact angle of TPU@Rc also reached 77.5 ± 5.7°, showing its hydrophilicity. The contact angle of TPU@Rd_Rc co-loaded with the two drug extracts further decreased to 65.6 ± 1.8° compared with TPU nanofibers loaded with a single drug, which revealed that the two drug extracts had a certain superposition effect in promoting the hydrophilic transition of TPU nanofibers. The contact angle of TPU@nAg#Rd_Rc with extra AgNP loading was 64.4 ± 2.9°, which was consistent with TPU@Rd_Rc. The loading of the two drug extracts improved the hydrophilicity of TPU nanofibers and the loaded AgNPs had no significant effect on the hydrophilicity of nanofibers. It was speculated that the co-loading of Rd and Rc could expedite the absorption of wound exudates by TPU nanofibers. Fig. S5E† showed that the SR of TPU@None, TPU@Rd, TPU@Rc, TPU@Rd_Rc and TPU@nAg#Rd_Rc after 24 h was separately 119.22 ± 4.23%, 159.46 ± 11.43%, 156.09 ± 2.19%, 171.25 ± 1.23% and 161.96 ± 2.62%. They all had good water absorption and could absorb wound exudates. Based on the healing theory of wet wounds, the ideal WVTR range is 2000–2500 g m^−2^ d^−1^.^49^ The experimental results revealed that the WVTR of TPU@None was 2925.9 ± 24.7 g m^−2^ d^−1^ and exceeded the ideal range, which might cause dryness of the wound surface and was not conducive to maintaining wounds wet and facilitating cell proliferation. However, the WVTR of TPU@Rd, TPU@Rc, TPU@Rd_Rc and TPU@nAg#Rd_Rc loaded with drugs was 2266.5 ± 105.4, 2254.7 ± 87.1, 2231.2 ± 17.3 and 2219.2 ± 86.2 g m^−2^ d^−1^, respectively (Fig. S5F†), and they were all within the ideal range. This ensured the timely absorption of wound exudates while maintaining the wound moisture, which had a positive significance for wound healing.^63^

### 3.4 Antioxidant property

The antioxidant and free-radical scavenging abilities of different TPU nanofiber membranes were assessed using the DPPH method. The higher clearance rate on DPPH displayed the stronger antioxidant capacity of nanofibers.^64^ As seen in Fig. S5G,† the clearance rate of TPU@None was only 4.95%, indicating that TPU materials did not have the ability to scavenge free radicals. After adding the drugs, there was a significant improvement in the clearance rates of TPU@Rd, TPU@Rc, TPU@Rd_Rc and TPU@nAg#Rd_Rc on DPPH at 30 min in comparison with TPU@None, showing the varying degrees of antioxidant activity. Among them, the clearance rate of TPU@Rc on DPPH was the highest and about 80% free radicals were cleared in 30 min, exhibiting significant antioxidant and free radical scavenging abilities. During the process of wound healing, excessive free radicals had adverse effects on wound healing and needed to be eliminated, but a small and moderate amount of free radical generation was necessary. The experimental results showed that TPU nanofiber membranes loaded with drugs could eliminate some free radicals while also retaining some free radicals so as to avoid the adverse effects of excessive production and accumulation of free radicals on wound healing, and activate immunity and cell proliferation, which played a certain promotional role in wound healing.^65^ Transition metal complexes had certain free radical scavenging activity and antioxidant property.^66^ Here, AgNPs might be more advantageous than ligands in balancing free radicals.

### 3.5 Blood compatibility and coagulability

To study the blood compatibility of TPU nanofibers, *in vitro* hemolysis experiments were conducted for evaluation. Fig. S5H† showed that the hemolysis rates of the five different TPU nanofiber membranes were all less than 5%, and hemolysis did not occur.^67^ As shown in Fig. S5I,† the supernatant of each TPU nanofiber sample was colorless or light yellow, implying the absence of hemoglobin and red blood cells in the supernatants. The above-mentioned results declared that the prepared TPU nanofibers had a relatively low hemolysis rate, indicating good blood compatibility.

In order to investigate the coagulation performance of different TPU nanofiber membranes, BCI and coagulation time were used for assessment. Fig. S5J† showed that the BCI of TPU@None significantly decreased compared to the blank group, effectively promoting coagulation. After loading drugs, the coagulation effects of TPU@Rd, TPU@Rc, TPU@Rd_Rc and TPU@nAg#Rd_Rc were further improved.

Fig. S5K† demonstrated that the coagulation time of TPU@None was significantly shortened compared with the blank group and the gauze, and those of TPU@Rd and TPU@Rc were also obviously shorter than that of TPU@None after drug loading. The photos of coagulation in each group at 5 min were also consistent with our experimental results (Fig. S5L†). The above results indicated that drug-loaded TPU nanofibers could effectively promote coagulation.

### 3.6 Antibacterial property

**Fig. 2:**
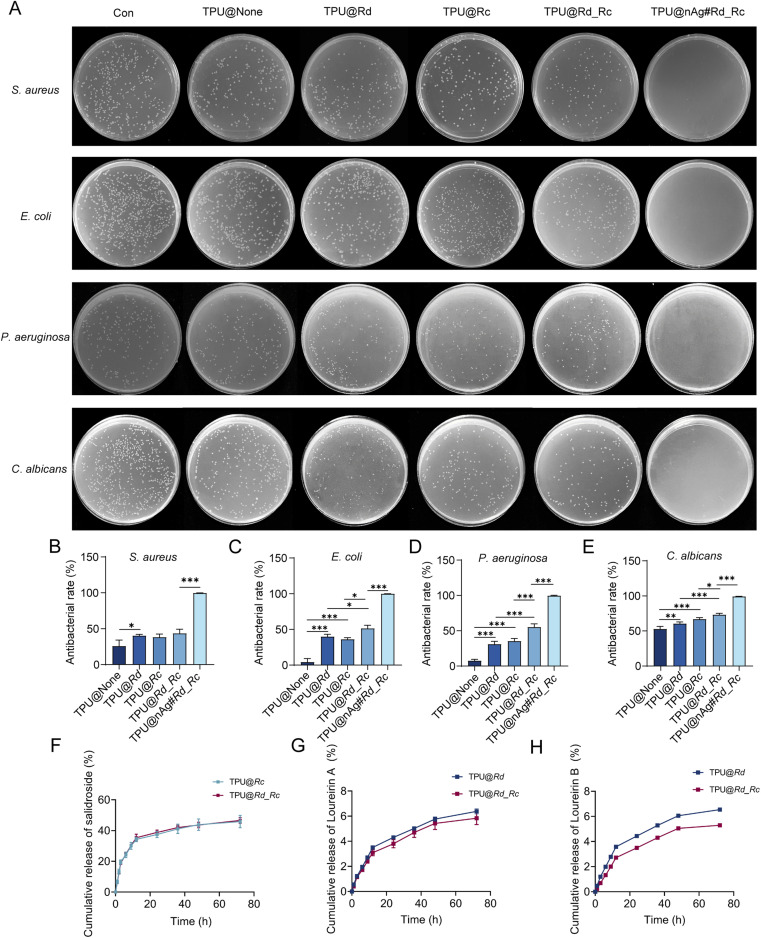
(A) Schematic diagram of each colony after treatment with nanofibers; (B) antibacterial rate of TPU nanofibers against *S. aureus*; (C) antibacterial rate of TPU nanofibers against *E. coli*; (D) antibacterial rate of TPU nanofibers against *P*. *aeruginosa*; (E) antibacterial rate of TPU nanofibers against *C. albicans*; (F) release curve of salidroside; (G) release curve of loureirin A; (H) release curve of loureirin B.

Common microorganisms causing wound infections include *P*. *aeruginosa*, *S. aureus*, *E. coli*, *Mycobacterium tuberculosis*, *Streptococcus pyogenes*, *Clostridium tetani* and some fungi.^68–72^*S. aureus*, *E. coli*, *P. aeruginosa* and *C. albicans* were chosen to evaluate the antibacterial effect of different TPU nanofiber membranes using the oscillation method. Fig. 2A showed that the antibacterial activity of TPU@Rd against *S. aureus* was sensibly higher than that of TPU@None. Meanwhile, TPU@nAg#Rd_Rc loaded with AgNPs revealed a more significant antibacterial property against *S. aureus* than TPU@Rd_Rc, and there was almost no growth of colonies in the plate (Fig. 2B). In the antibacterial experiments against *E. coli*, *P. aeruginosa* and *C. albicans* (Fig. 2C–E), similar experimental results were obtained. The antibacterial ability of TPU@Rd and TPU@Rc loaded with only one drug was better than that of TPU@None. TPU@Rd_Rc loaded with two drugs showed dramatically better antibacterial activity than TPU@Rd and TPU@Rc. The antibacterial effect of TPU@nAg#Rd_Rc loaded with AgNPs was obviously better than that of TPU@Rd_Rc, showing almost no growth of colonies. These results declared that Rd and Rc had a certain antibacterial performance, and the antibacterial effect of nanofibers simultaneously co-loaded with two drugs was stronger than that of nanofibers loaded with a single drug, but the antibacterial ability of AgNPs with Rd and Rc was the strongest. This was consistent with the physiological functions of transition metal complexes mentioned in the literature.^66,73–75^

### 3.7 Release behaviour

The drug release behaviour of nanofiber membranes was studied by HPLC using salidroside with loureirin A and loureirin B as the indices. Fig. S6† shows the peak plots of the standard samples, Rd and Rc. Fig. 2F displayed that the release of salidroside from TPU@Rc and TPU@Rd_Rc was rapid in the first 12 h and the cumulative release reached about 35%. The drug continuously released from the nanofibers and the release rate gradually slowed down from 12 h to 72 h. After 72 h, the cumulative release of salidroside in the two materials reached about 45%, indicating that the drug could continuously release without replacing wound dressings for a long time. There was no obvious difference in the release rate of salidroside between TPU@Rc and TPU@Rd_Rc, which demonstrated that Rd did not affect the release behaviour of salidroside. Due to the similarity in structure of loureirin A and loureirin B, their release behaviour exhibited similar curves (Fig. 2G and H). From 0 h to 12 h, loureirin A and loureirin B rapidly released to reach the cumulative release of about 3%. As the time went by, loureirin A and loureirin B began to slowly and continuously release from 12 h to 72 h, and their release rate was about 6% after 72 h. The release of the two effective components had not yet reached the plateau period as seen from the release curves. The two active ingredients could still release slowly and continuously with time. Additionally, according to the cumulative release rate, it could be concluded that the binding force between loureirin A, loureirin B and nanofibers was stronger than salidroside, and the burst-release effect was weaker than salidroside, illustrating the better controlled-release effect of loureirin A and loureirin B. Because of the better hydrophilicity of monosaccharides and phenolic hydroxyl groups in salidroside, it tended to release into water. But loureirin A and loureirin B also contained the benzyl ether structure, exhibiting certain hydrophobicity, so they released more slowly than salidroside. The results above showed that the manufactured TPU nanofibers could rapidly release drugs in the first 12 h. However, the drugs slowly continued to release from nanofibers to achieve controlled release after 12 h. This could not only alleviate wound symptoms, but also release drugs even without replacing wound dressings for a long time, which could continuously promote wound healing and avoid the secondary injuries caused by multiple changes of wound dressings.

### 3.8 Biocompatibility and cell migration

**Fig. 3:**
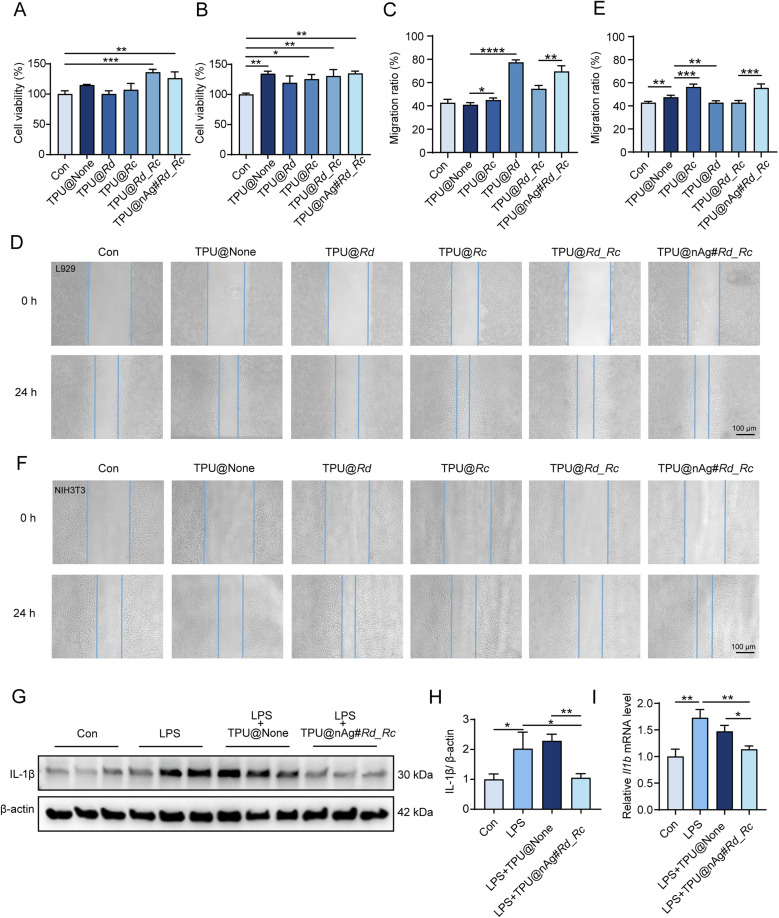
Biocompatibility and anti-inflammatory properties of TPU nanofibers. (A) Statistical chart of L929 cell viability; (B) statistical chart of NIH3T3 cell viability; (C) statistical chart of L929 cell migration rate; (D) statistical chart of NIH3T3 cell migration rate; (E) L929 cell migration map; (F) NIH3T3 cell migration map; (G) western blot detection of IL-1β expression levels in NIH3T3 cells with different treatments after LPS induction; (H) statistical chart of IL-1β expression levels; (I) expression levels of Il1b in NIH3T3 cells with different treatments after LPS induction.

MTT assay was employed to evaluate the cytotoxicity of leachates from TPU nanofiber membranes on mouse epithelial-like fibroblast cells (L929) and mouse embryonic fibroblast cells (NIH3T3). As depicted in Fig. 3A, the viability of L929 cells treated with the TPU@None leachate showed no significant difference compared to the control group. However, L929 cells treated with the TPU@Rd_Rc leachate containing Rd and Rc, and TPU@nAg#Rd_Rc leachate containing AgNPs demonstrated a significant increase in cell viability. This suggested that the co-loading of these extracts of Chinese medicinal materials effectively enhanced the proliferation of L929 cells. Notably, the cell viability in the TPU@nAg#Rd_Rc group showed no statistical difference from the TPU@Rd_Rc group, indicating that the addition of AgNPs did not adversely affect the biosafety of TPU nanofibers. The MTT results for NIH3T3 cells (Fig. 3B) revealed that cell viability in the TPU@None, TPU@Rc, TPU@Rd_Rc and TPU@nAg#Rd_Rc groups was obviously higher than that in the control group. This demonstrated that our fabricated TPU nanofibers significantly enhanced the vitality of NIH3T3 cells, promoting cell proliferation, and the incorporation of AgNPs did not compromise their biosafety. Overall, these results demonstrated that the TPU nanofibers we prepared possessed excellent biosafety properties. The loading of Rd and Rc effectively boosted cell vitality and facilitated cell proliferation.

To investigate the impact of TPU nanofiber membranes on the *in vitro* migration of fibroblasts, the cell scratch assay was conducted using L929 and NIH3T3 cells to assess the influence of nanofiber membranes on wound healing. The scratch assay results for L929 cells (Fig. 3C and D) showed that the TPU@None group without adding extracts had no statistical difference in cell migration after 24 h compared to the control group. The groups treated with TPU nanofiber membranes loaded with Rd and Rc exhibited a significant reduction in scratch area in comparison with the TPU@None group, implying a marked increase in cell migration rate. The co-loading of these extracts effectively enhanced the migration rate of L929 cells. Furthermore, the cell migration rate in the TPU@nAg#Rd_Rc group loaded with AgNPs was sensibly higher than that in the TPU@Rd_Rc group without AgNPs, suggesting a positive effect of AgNP loading on L929 cell migration.^76^ The cell migration results for NIH3T3 cells (Fig. 3E and F) revealed that the blank TPU group showed a notable increase in migration rate compared to the control group, and NIH3T3 cells treated with the TPU@Rd group also had a markedly higher migration rate than those treated with the TPU@None group, indicating that Rd effectively promoted NIH3T3 cell migration. Similarly, the migration rate of the TPU@nAg#Rd_Rc group loaded with AgNPs was significantly higher than that of the TPU@Rd_Rc group. These results demonstrated that TPU@nAg#Rd_Rc could effectively enhance cell migration and accelerate wound healing.

### 3.9 Anti-inflammatory effect

Anti-inflammation plays a crucial role in expediting wound healing in diabetic conditions. The transcription and expression changes of inflammatory cytokines were assessed by treating NIH3T3 cells induced by LPS with leachates from TPU@None and TPU@nAg#Rd_Rc. Western blot analysis (Fig. 3G) revealed that IL-1β levels in NIH3T3 cells obviously increased after LPS treatment, confirming the successful establishment of an LPS-induced inflammation model. Post treatment with the TPU@nAg#Rd_Rc leachate, there was a significant reduction in IL-1β expression in NIH3T3 cells compared with both the model group and TPU@None leachate group (Fig. 3H). Further, the results of qPCR experiments (Fig. 3I) displayed that the transcription level of *Il1b* in NIH3T3 cells was significantly reduced after treatment with the TPU@nAg#Rd_Rc leachate in comparison to the model and TPU@None leachate groups. These results declared that TPU@nAg#Rd_Rc could effectively lower the levels of inflammatory cytokines in cells, thus exhibiting anti-inflammatory properties.

### 3.10 Ability to promote wound healing in diabetic mice

#### 3.10.1 Wound healing in diabetic mice

**Fig. 4:**
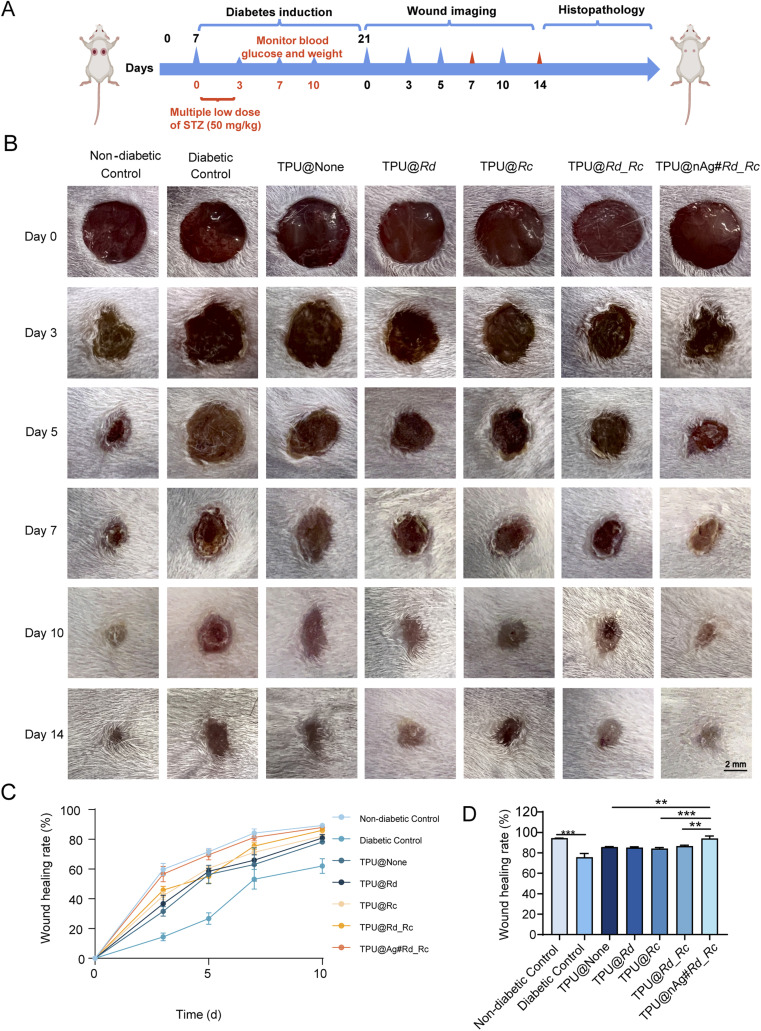
Wound healing of diabetic mice after different treatments. (A) The flow chart of wound model establishment in diabetic mice; (B) wound pictures of diabetic mice after treatments with different TPU nanofibers on days 0, 3, 5, 7, 10 and 14; (C) wound healing rates of diabetic mice on days 0, 3, 5, 7, and 10 after treated with different TPU nanofibers; (D) wound healing rates of diabetic mice on the 14th day after treatments with different TPU nanofibers.

Cellular-level studies showed that TPU@None, TPU@Rd, TPU@Rc, TPU@Rd_Rc and TPU@nAg#Rd_Rc exhibited no significant cytotoxicity and the incorporation of AgNPs into TPU nanofiber membranes positively influenced the proliferation and migration of NIH3T3 and L929 cells. In diabetic mouse models, we evaluated whether these nanofiber membranes could enhance diabetic wound healing (Fig. 4A). The wound healing images (Fig. 4B) and statistical results (Fig. 4C) showed that the healthy control group had smaller wounds and higher healing rates than the other groups on the third day after treatment with nanofiber membranes. The wound area in the diabetic control group was the largest and its healing rate was the lowest, only 14.40 ± 2.14%, declaring that the healing speed of diabetic wounds was significantly lower than that of normal wounds without therapeutic intervention and it was 59.78 ± 3.25%. Moreover, the wound healing rate in the TPU@None treatment group was 31.55 ± 2.60%, while TPU@Rd, TPU@Rc, TPU@Rd_Rc, and TPU@nAg#Rd_Rc treatment groups showed healing rates of 36.71 ± 4.61%, 42.04 ± 2.19%, 45.85 ± 2.06% and 56.59 ± 4.28%. By the 14th day, the results displayed that the wound healing rate in the healthy control group reached 89.27 ± 0.83% and the TPU@nAg#Rd_Rc treatment group achieved a healing rate of 87.92 ± 0.82%, closely matching the healthy control group and dramatically exceeding the other TPU treatment groups (Fig. 4D).

In contrast, the diabetic control group had the lowest healing rate, only 62.00 ± 4.02%. These findings indicated that TPU nanofiber wound dressing loaded with AgNPs could mitigate the adverse effects of diabetes on wound healing and effectively promote wound closure in diabetic mice.

#### 3.10.2 Epidermal regeneration and collagen remodeling

**Fig. 5:**
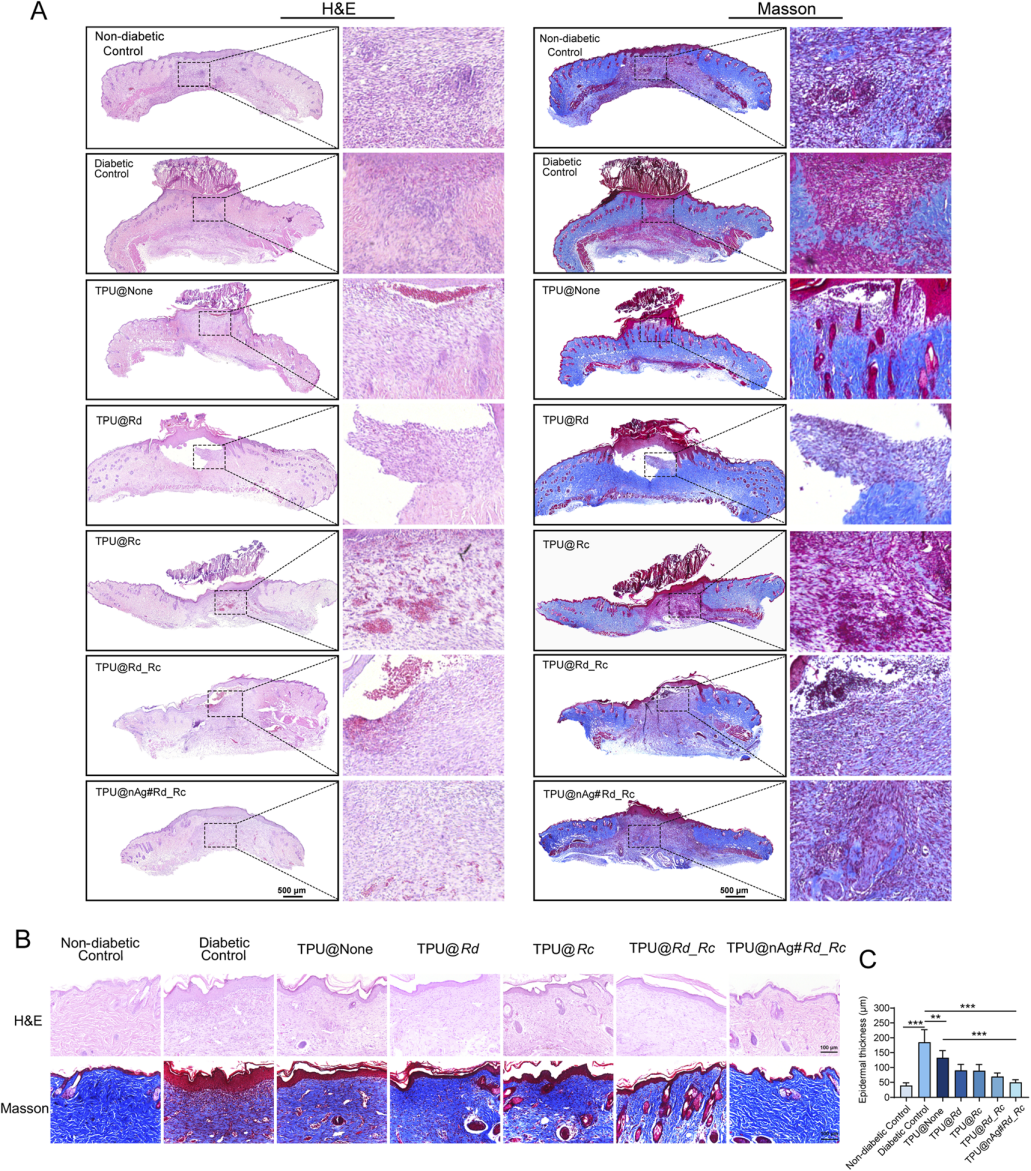
Skin tissue staining of wounds in diabetic mice. (A) H&E and Masson staining of skin tissue in wounds of diabetic mice treated with different TPU nanofibers after 7 days; (B) H&E and Masson staining of skin tissue in wounds of diabetic mice treated with different TPU nanofibers after 14 days; (C) epidermal thickness of skin tissues in wounds of diabetic mice treated with different TPU nanofibers after 14 days.

H&E and Masson staining were used to assess the epidermal regeneration and collagen remodeling of diabetic mouse wounds following different treatments. The H&E staining results showed that the wounds in the diabetic control group, TPU@None, TPU@Rd and TPU@Rc treatment groups showed poor healing on the 7th day with incomplete epidermal repair and noticeable irregular scabbing (Fig. 5A). The TPU@Rd_Rc group showed more intact epidermis, but granulation tissue was poorly repaired with subcutaneous bleeding and a loose structure. The healthy control and TPU@nAg#Rd_Rc treatment groups had intact and compact epidermal structures with thicker granulation tissue formation. On the 14th day (Fig. 5B), all groups exhibited epidermal regeneration, but the diabetic control group, TPU@None, TPU@Rd, and TPU@Rc treatment groups had significant inflammatory cell infiltration and thicker epidermis at the wound site, suggesting that premature and irregular scabbing without effective control of inflammation led to the accumulation of blood and fluid under the scab and induced inflammation that did not heal and worsened. In contrast, the healthy control, TPU@Rd_Rc and TPU@nAg#Rd_Rc treatment groups had less inflammatory cell infiltration. The epidermal thickness statistics (Fig. 5C) also showed that the epidermis in the TPU@nAg#Rd_Rc treatment group was mature and comparable in thickness to the healthy control group, manifesting that the loading of AgNPs significantly promoted the maturation of wound epidermis.

Masson staining was performed to observe collagen deposition in diabetic mouse wounds treated with different TPU nanofiber dressings on day 7 (Fig. 5A), and the results were similar to those observed with H&E staining. At this stage, the healthy control, TPU@Rd_Rc and TPU@nAg#Rd_Rc treatment groups showed a small amount of collagen deposition in the center of wounds, while collagen deposition was not evident in the other groups. This declared that the wounds in healthy control, TPU@Rd_Rc and TPU@nAg#Rd_Rc treatment groups had entered the repair phase by day 7, while the other groups were still in the inflammatory phase. All treatment groups exhibited collagen fiber deposition on day 14 (Fig. 5B). However, the diabetic control group, TPU@None, TPU@Rd and TPU@Rc treatment groups showed low level collagen deposition without structure in the wound area, while the healthy control group and TPU@nAg#Rd_Rc treatment group displayed increased collagen deposition characterized by loose collagen fibers. These results displayed that the treatment with TPU@nAg#Rd_Rc promoted epidermal remodeling, granulation tissue repair and collagen fiber deposition in diabetic mouse wounds, facilitating wound healing. TPU@nAg#Rd_Rc treatment accelerated diabetic mouse wound healing by enhancing epidermal maturation and the deposition of mature bundles of collagen fibers in the wound area.

#### 3.10.3 Anti-inflammation

**Fig. 6:**
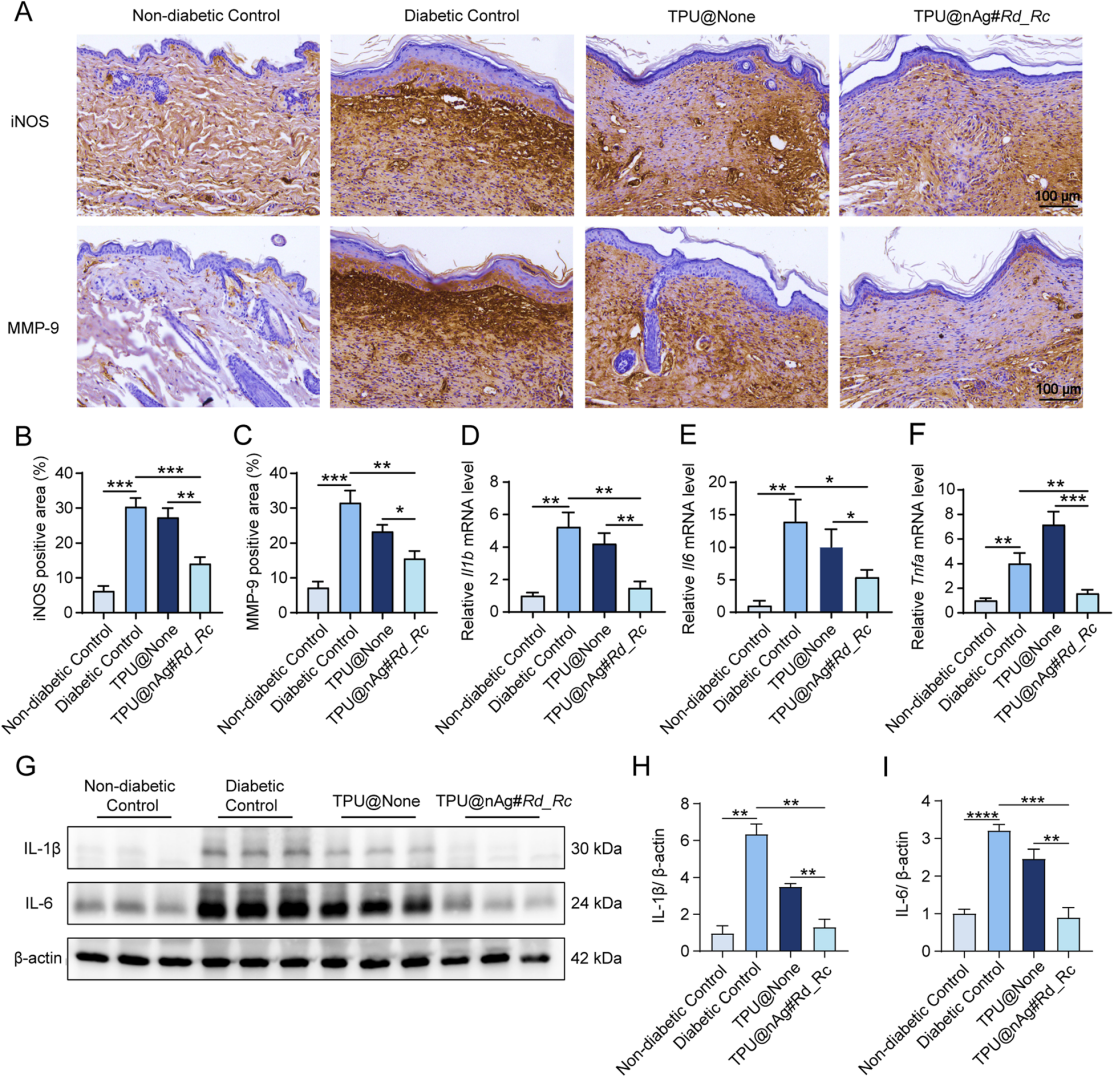
Inflammation in diabetic wounds reduced by TPU nanofibers. (A) Expression levels of iNOS and MMP-9 in wounds of diabetic mice with different treatments for 7 days; (B) percentage of iNOS positive regions; (C) percentage of MMP-9 positive regions; (D) *Il1b* expression level in wounds of diabetic mice with different treatments for 7 days; (E) *Il6* expression level in wounds of diabetic mice with different treatments for 7 days; (F) *Tnfa* expression level in wounds of diabetic mice with different treatments for 7 days; (G) IL-1β and IL-6 expression levels in wounds of diabetic mice with different treatments for 7 days by western blot detection; (H) statistical chart of IL-1β expression level; (I) statistical chart of IL-6 expression level.

In chronic diabetic wounds, pro-inflammatory macrophages secrete inflammatory mediators such as iNOS, which has a negative impact on the wound microenvironment at high concentrations.^77^ Therefore, the expression levels of iNOS in wound tissues of diabetic mice in each treatment group were assessed through iNOS immunohistochemical staining. The results showed that the diabetic control group exhibited an obvious increase in iNOS-positive area in comparison with the healthy control group (Fig. 6A). However, the iNOS-positive area was markedly reduced after treatment with TPU@nAg#Rd_Rc compared to both the diabetic control group and TPU@None treatment group (Fig. 6B).

MMP-9 is a matrix metalloproteinase involved in the degradation and remodeling of collagen during the wound healing process.^78^ In chronic diabetic wounds, excessive activation of MMP-9 leads to the degradation of local ECM.^79^ Cell migration is impaired when there is a lack of ECM scaffold, hindering the closure of wound epithelium and increasing the risk of infection. Excessive MMP-9 has been identified as a major pathogenic factor in delaying diabetic wound healing.^13,80,81^ Therefore, MMP-9 immunohistochemical staining was performed to observe MMP-9 expression levels in wound tissues of diabetic mice in each treatment group. The results declared that the diabetic control group exhibited a significant increase in MMP-9-positive area compared to the healthy control group (Fig. 6A). However, after treatment with TPU@nAg#Rd_Rc, the MMP-9-positive area was markedly reduced in comparison with both the diabetic control group and TPU@None treatment group (Fig. 6C).

To assess the ability of TPU nanofibers to reduce inflammation levels at diabetic wound sites, the transcription and expression levels of inflammatory factors in wound tissues treated with different interventions for 7 days were measured. The results displayed that the expressions of *Il1b*, *Il6* and *Tnfa* in the diabetic control group were obviously upregulated compared to the healthy control group, indicating a higher level of inflammation at the diabetic wound sites (Fig. 6D–F). After treatment with TPU@nAg#Rd_Rc, expression levels of *Il1b*, *Il6* and *Tnfa* in the wound tissues of diabetic mice were significantly downregulated in comparison with both the diabetic control group and TPU@None treatment group. These results demonstrated that TPU@nAg#Rd_Rc effectively reduced the expression of inflammatory factors at diabetic wound sites, achieving an anti-inflammatory effect.

Western blot analysis was performed to assess the changes in expressions of IL-1β and IL-6 in mouse wound tissues after different treatments for 7 days (Fig. 6G). The results revealed that expression levels of IL-1β and IL-6 in the diabetic control group were significantly upregulated compared to the healthy control group, representing a pronounced increase in inflammation. In comparison with both the diabetic control group and TPU@None treatment group, IL-1β and IL-6 expression levels in wound tissues of diabetic mice were markedly downregulated after treatment with TPU@nAg#Rd_Rc (Fig. 6H and I). These findings were consistent with the results obtained from qPCR analysis (Fig. 6D and E).

The above results showed that TPU@nAg#Rd_Rc could effectively alleviate the inflammatory reaction of diabetic mice, shorten the wound inflammatory period and inhibit collagen degradation, thus accelerating the wound healing of diabetic wounds.

#### 3.10.4 Anti-lipid peroxidation

To evaluate whether TPU nanofibers had the ability to reduce LPO levels at the wound site, MDA detection was firstly conducted at the NIH3T3 cell level. The results showed that MDA levels of NIH3T3 cells were significantly improved after LPS induction, but their MDA level was obviously decreased after being treated with TPU@nAg#Rd_Rc (Fig. S7A†). This implied that TPU@nAg#Rd_Rc could effectively reduce the LPO level of cells induced by LPS. Furthermore, MDA contents in the wound of diabetic mice were also detected after 7 and 14 days of different treatments. The results declared that the LPO level in the diabetic control group was significantly improved in comparison with the healthy control group after different treatments for 7 days (Fig. S7B†). The LOP levels, namely malondialdehyde concentration obviously decreased after the treatment with TPU@None, TPU@nAg#Rd_Rc and the LPO level was lower than TPU@None after treated with TPU@nAg#Rd_Rc. After 14 days of treatments (Fig. S7C†), MDA content in the wound skin of diabetic mice treated with TPU@nAg#Rd_Rc was also significantly lower than that of the diabetic control group and TPU@None treatment group. All the above results declared that TPU@nAg#Rd_Rc could facilitate the healing of diabetic wounds by significantly reducing the LPO level of diabetic wounds.

#### 3.10.5 Angiogenesis

Angiogenesis is one of the key processes in wound healing. The formation of new blood vessels offered oxygen and nutrients to the wounds, assisting the growth and differentiation of neonatal cells.^82^ However, the angiogenesis ability of diabetic patients is inhibited, leading to ischemia and malnutrition for wounds, further delaying wound healing.^83,84^ VEGF is a vascular endothelial growth factor that can redound to the generation and regeneration of new blood vessels.^85^ The expression level of VEGF is usually reduced in diabetic wound healing, bringing out the reduced angiogenesis and insufficient blood supply, thus affecting wound repair.^86^ Therefore, the effects of TPU nanofibers on the VEGF expression level in the tissue of diabetic wounds were measured to evaluate the vascular regeneration at the wound site (Fig. S7D†). The results showed that the expression level of VEGF in the diabetic control group was significantly decreased compared with the healthy control group (Fig. S7E†). After the treatment of TPU@nAg#Rd_Rc, VEGF expression level was obviously higher than that of the diabetic control group and TPU@None treatment group (Fig. S7E†), which explained that TPU@nAg#Rd_Rc could effectively advance the VEGF expression in diabetic wounds, thereby facilitating vascular regeneration. The above-mentioned results expressed that TPU@nAg#Rd_Rc could expedite the healing of diabetic wounds by promoting angiogenesis.

## 4 Conclusions

The TPU@nAg#Rd_Rc nanofiber membrane, a kind of wound dressing used to accelerate diabetic wound healing, was fabricated by *in situ* construction and loading AgNPs. AgNPs not only had an antibacterial property and free radical scavenging activity, but also could promote the migration and proliferation of fibroblasts, alleviate the inflammatory reaction of wounds, reduce the LPO level and assist angiogenesis, thus facilitating the healing of diabetic wounds. Owing to the excellent mechanical properties of the TPU material itself, this wound dressing exhibited good elasticity and toughness, which could satisfy the mechanical needs of diabetic wound reconstruction. The addition of Rd and Rc improved the water absorption and air permeability of TPU nanofibers, which could both uptake wound exudates in time and ensure that the wounds were in a humid environment, helping the healing of diabetic wounds. Moreover, the wound healing rate of the treatment group treated with TPU@nAg#Rd_Rc reached 87.92% ± 0.82% in the wound model of diabetic mice 14 days later, and epidermal remodeling and collagen deposition were in a good state, similar to the healthy control group. The results also proved that TPU@nAg#Rd_Rc might accelerate diabetic wound healing by promoting VEGF expression, while inhibiting LPO and the expressions of IL-6, IL-1B, MMP-9 and iNOS, which displayed the potential for the treatment of diabetic wounds and skin tissue regeneration. As the loaded drug, AgNPs with Rd and Rc would provide a new strategy for the treatment of diseases related to oxidative stress and inflammatory abnormality or self-repair.

## Data availability

The data underlying this article are available in the article and its online ESI.†

## Conflicts of interest

There are no conflicts to declare.

## Supplementary Material

RA-014-D4RA04860A-s001
