# Supplementary material for: Multifunctional combined drug-loaded nanofibrous dressings with anti-inflammatory, antioxidant stress and microenvironment improvement for diabetic wounds
Source: RSC Adv. 2024 Sep 18;14(40):29606–23. doi: 10.1039/d4ra04860a (PMC11409454; doi:10.1039/d4ra04860a)
Supplement: RA-014-D4RA04860A-s001 [file RA-014-D4RA04860A-s001.pdf]

Supplementary Information (SI) for RSC Advances.
This journal is © The Royal Society of Chemistry 2024

Supplementary information

Multifunctional combined drug-loaded nanofibrous dressings with anti-inflammatory, antioxidant stress and microenvironment improvement for diabetic wounds

Yuqing Ju[a, b], Yuxuan Luo[c, d], Ruimeng Li[a, b], Wei Zhang[c, d], Yan Ge[c, d], Jiapeng Tang[a, b] *

[a.] Institute of Special Environmental Medicine, Nantong University, Nantong 226019, PR China.

[b.] Co-innovation Center of Neuroregeneration, Nantong University, Nantong 226001, PR China.

[c.] School of Textile and Clothing, Nantong University, Nantong 226019, PR China.

[d.] National & Local Joint Engineering Research Center of Technical Fiber Composites for Safety and Protection, Nantong University, Nantong 226019, PR China.

*Correspondence to: Jiapeng Tang

Institute of Special Environmental Medicine, Nantong University

No. 9, Seyuan Road, Chongchuan District, Nantong City, Jiangsu Province, China

Tel.: +86-513-55003378

Fax: +86-513-55003378

Email: jptang@ntu.edu.cn

Methods

HPLC analysis of active ingredients in pharmaceutical extracts

Loureirin A, loureirin B and salidroside were selected as representative active components of Resina Draconis and Rhodiola rosea L. extracts. Chromatographic conditions: The column (Nucifera C18P 12 nm 5 μm, 4.6 mm×250 mm), mobile phase A was 0.1% acetic acid, and B was acetonitrile. The elution profile was as follows: 0 min: 90% A, 10% B; 0-20 min: 60% A, 40% B; 20-55 min: 60% A, 40% B; 55-58 min: 90% A, 10% B; 58-60 min: 90% A, 10% B. The flow rate was 1 ml/min, the injection volume was 10 μl, the column temperature was 30 ℃, and ultraviolet detection wavelength was 270 nm.

Western blot

The protein extracted from cells and mouse wounds was quantified using the bicinchoninic acid assay (BCA) to calculate the protein concentration. Proteins were then separated by SDS-PAGE and transferred onto PVDF membranes. The membranes were blocked using 5% non-fat milk powder and incubated overnight at 4°C with primary antibodies, including anti-IL-1β (Abcam, ab9722), anti-β-actin (Sigma, A5316), Anti-IL6 (Abcam, ab9324), and Anti-VEGF (Millipore, 2688591). The binding of the primary antibodies was visualized using a goat anti-rabbit HRP-conjugated secondary antibody (Jason, 115-035-033) and a goat anti-mouse HRP-conjugated secondary antibody (Jason, 111-035-003). A chemiluminescence imaging system (4100, Tanon, China) was used for visualization, and Image J software (version 1.5.2) was utilized for the analysis of the results.

qRT-PCR

Total RNA was isolated from the cells and tissue, and reverse-transcribed into cDNA using the HiScript®III RT SuperMix kit (Vazyme, R323-01), following the manufacturer's instructions. qRT-PCR primers were designed based on the NCBI mouse mRNA sequence database (Table. S3). After primer synthesis, the RT-PCR reaction system was prepared using the SYBR qPCR Master Mix (Vazyme, Q141-02). The prepared mixture was then loaded into a fluorescence quantitative PCR instrument (Step One, ABI, USA). Relative gene expression was calculated using the ΔΔCt method

for normalization to the reference gene.

MDA assay for measuring lipid peroxidation levels

After introducing the cells and mouse wound tissue into the extraction solution, ultrasonication or homogenization was employed for disruption. Subsequently, the resulting mixture was subjected to centrifugation, and the supernatant was carefully collected and stored at low temperatures (on ice) for subsequent analysis in our research study. The MDA assay was conducted according to the instructions provided in the MDA kit (BC0025, Solarbio, China) manual. The absorbances of each sample at 532 nm and 600 nm were measured using a microplate reader (Synergy2, BioTek Instruments, USA). The MDA content was calculated using the formula provided in the kit's manual.

H&E staining

Paraffin-embedded tissue sections were stained using the HE staining kit (C0105S, Beyotime, China). Paraffin-embedded tissue sections were deparaffinized with xylene, washed with gradient ethanol, and then rinsed with distilled water. The tissue sections were subsequently stained with hematoxylin and eosin solutions. After staining, the sections were dehydrated with a graded series of ethanol, rendered transparent with xylene treatment, and sealed with neutral resin. Finally, the tissue sections were examined under a microscope (DM4000B, Leica, Germany) for histological analysis.

Masson's trichrome staining

Paraffin-embedded tissue sections were stained using Masson's Trichrome stain kit (G1346, Solarbio, China). Following routine dewax to distilled water of paraffin-embedded tissue sections and subsequent washing with water, a series of staining solutions were applied sequentially. This included staining with Celestite Blue Solutio, Mayer Hematoxylin Solution, Acid Differentiation Solution, Ponceau-Acid Fuchsin solution, Phosphomolybdic Acid Solution, and Aniline Blue Solution followed by treatment with a weak acid solution. Subsequently, tissue sections were dehydrated with ethanol, rendered transparent with xylene and sealed with neutral resin for observation under an optical microscope (DM4000B, Leica, Germany).

Immunohistochemical staining

Immunohistochemical staining was performed to assess the expression levels of MMP9 and iNOS in mouse skin tissue. Paraffin-embedded sections were deparaffinized to distilled water and subjected to antigen retrieval using sodium citrate buffer, and the tissue boundaries were outlined with a hydrophobic pen. Endogenous peroxidase activity was blocked, and after rinsing with PBS, an anti-MMP9 antibody (Abcam, ab283575) and an anti-iNOS antibody (Abcam, ab283655) were added and incubated overnight at 4°C. Following this, tissue sections were incubated with secondary antibodies at room temperature after the addition of a reaction enhancer, and a DAB chromogenic solution was applied. Cell nuclei were stained with hematoxylin solution. Finally, tissue sections were dehydrated with a graded series of ethanol, rendered transparent with xylene, sealed with neutral resin, and observed under an optical microscope (DM4000B, Leica, Germany). The positive areas in the tissue were quantified using Image J (version 1.5.2).

Results

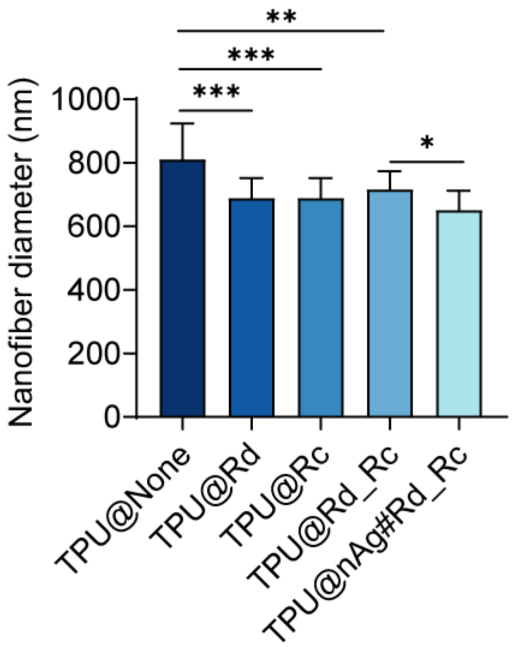

Fig. S1. Nanofiber diameter statistics.

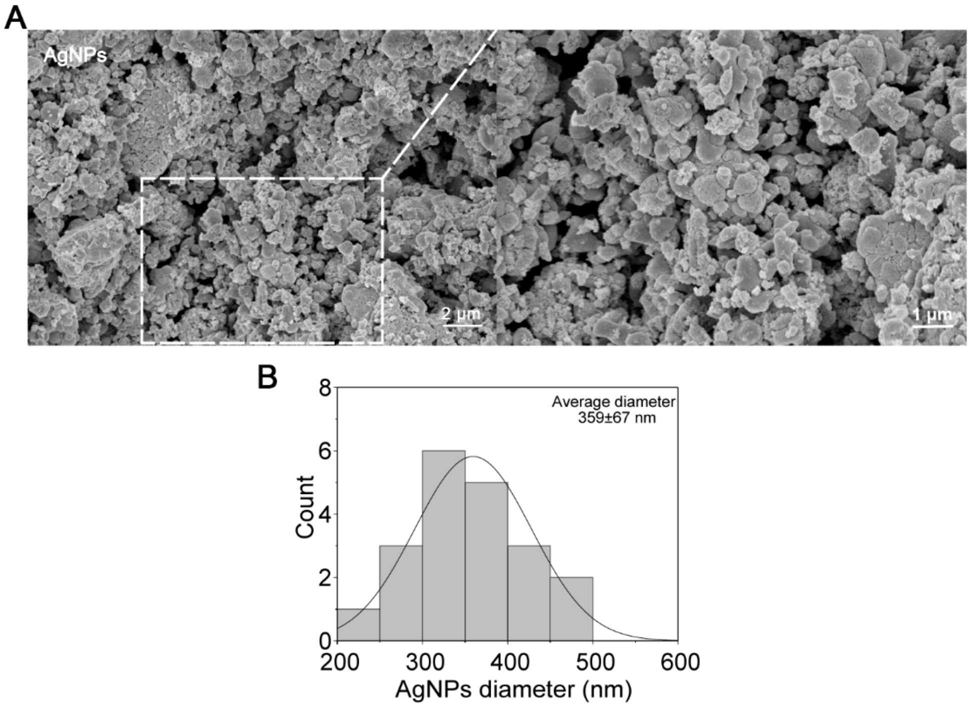

Fig. S2. The diameter of AgNPs. A) SEM image of AgNPs; B) Diameter statistics of AgNPs.

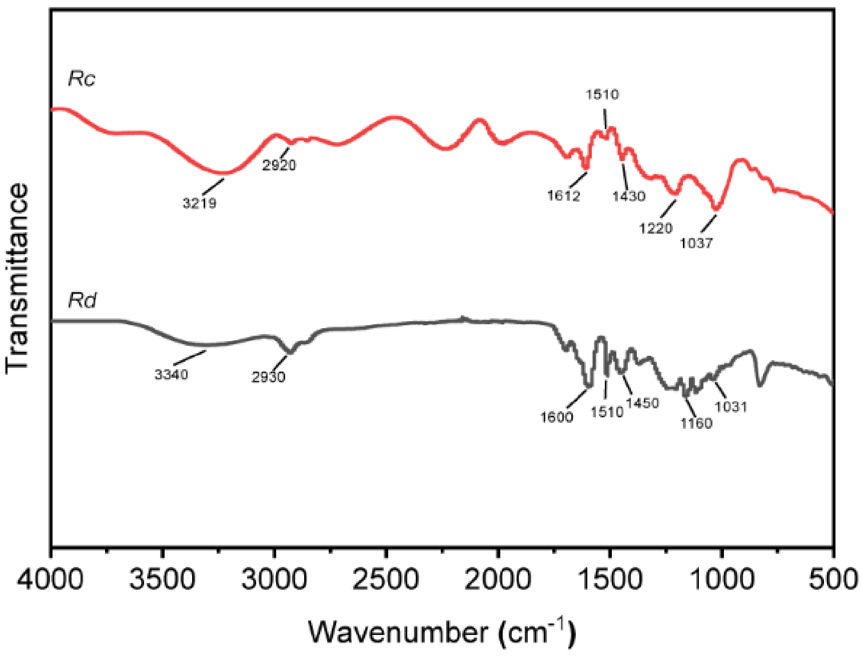

Fig. S3. FTIR of extracts from Resina Draconis and Rhodiola rosea L.

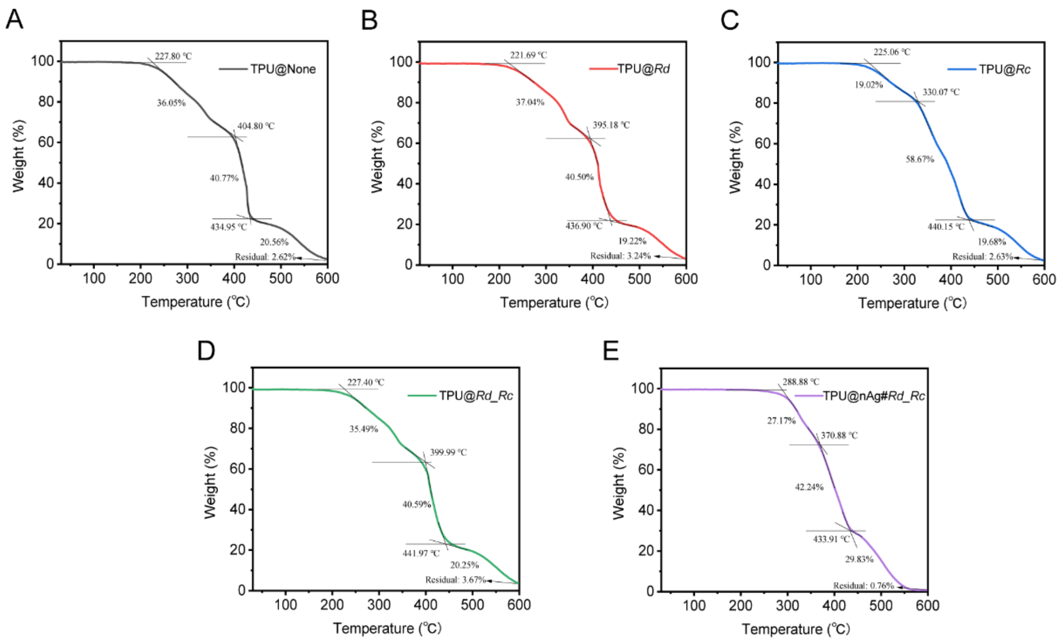

Fig. S4. TGA analysis diagram of TPU nanofibers.

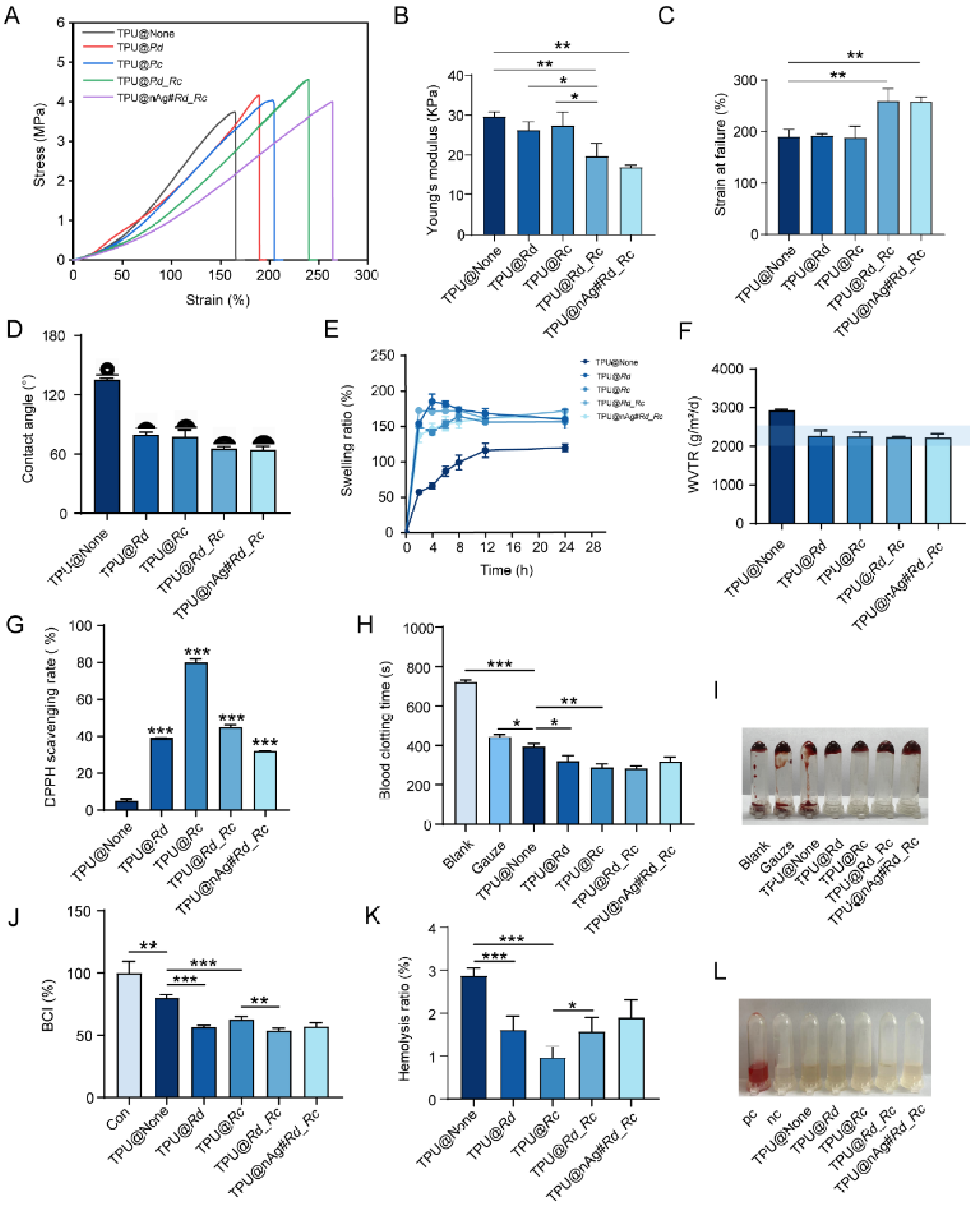

Fig. S5. A) Stress-strain curves of TPU nanofibers; B) Young's modulus of TPU nanofibers; C) Elongation at break of TPU nanofibers; D) Swelling rate of TPU nanofibers; E) Contact angle of TPU nanofibers; F) WVTR of TPU nanofibers; G) DPPH clearance rate of TPU nanofibers; H) Hemolysis rate of TPU nanofibers; I) Representative display pictures for hemolysis rate of TPU nanofibers. (pc represented the positive control, nc represented the negative control); J) BCI of TPU nanofibers; K) Coagulation time of TPU nanofibers; L) Coagulation effect of TPU nanofibers in 5 min.

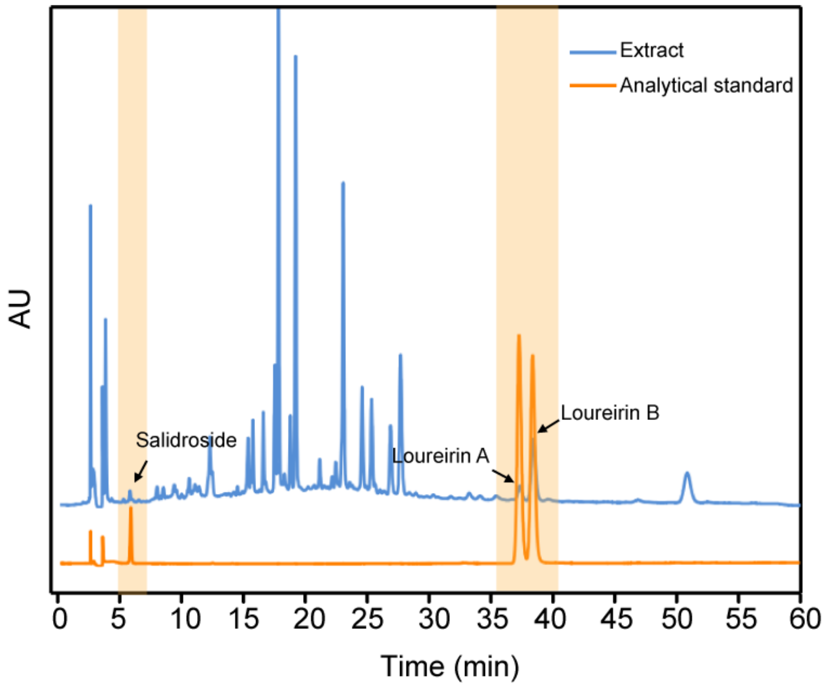

Fig. S6. HPLC chromatogram of a mixed solution of analytical standard (0.6 mg/ml salidroside, 0.5 mg/ml loureirin A, and 0.5 mg/ml loureirin B) and the solution containing 5 mg/ml Resina Draconis extract and 5 mg/ml Rhodiola rosea L. extract

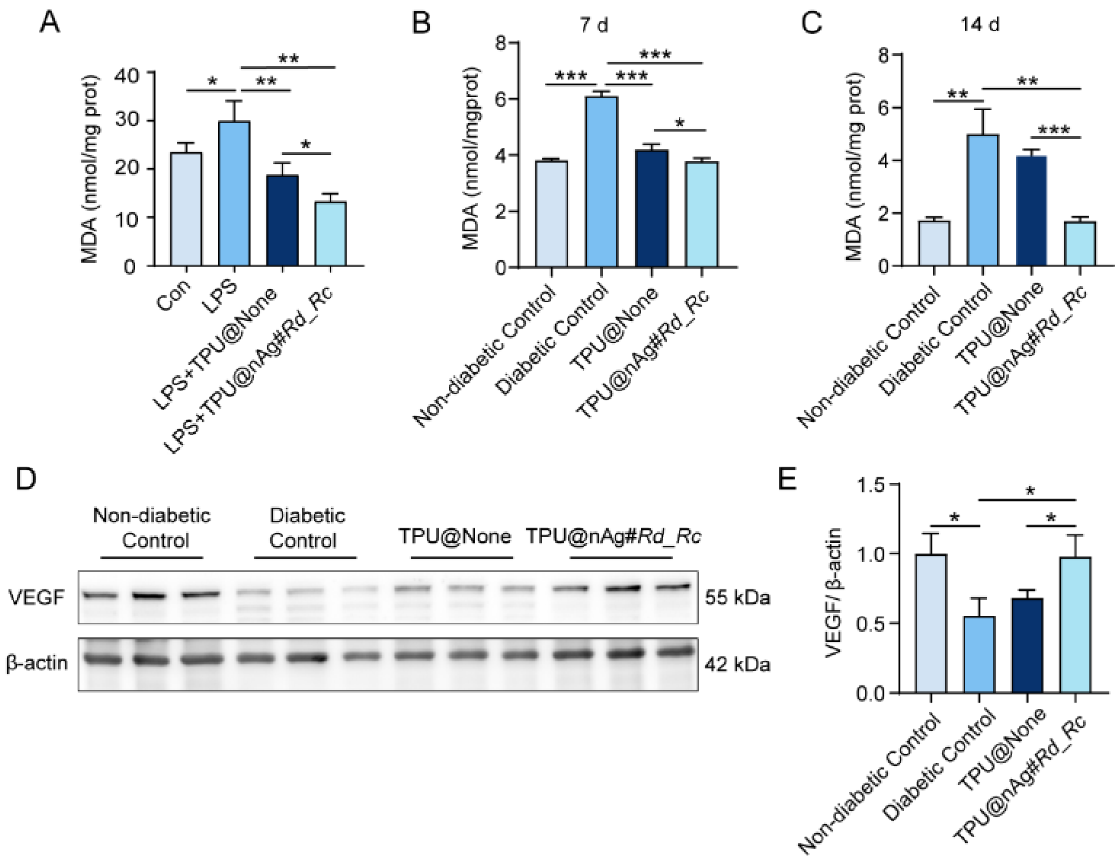

Fig. S7. TPU nanofibers reduce LPO and enhance vascular regeneration capabilities. A) MDA level in NIH3T3 cells induced by LPS; B) MDA content in wounds of diabetic mice with different treatments for 7 d; C) MDA content in wounds of diabetic mice with different treatments for 14 d; D) VEGF expression levels in wounds of diabetic mice with different treatments for 7 d by Western blot detection; E) Statistical chart of VEGF expression level.

Table S1. Calculation results of element content in the samples based on the XPS survey spectra.

Samples	Element content (atomic %)			
	C1s	O1s	N1s	Ag3d
TPU@none	72.81	23.11	3.48	0
TPU@Rd_Rc	74.10	22.52	2.83	0
TPU@nAg	78.74	16.92	3.49	0.31
TPU@nAg#Rd_Rc	69.26	24.79	4.82	0.32

Table S2. Thermodynamic properties of TPU nanofibers.

Samples	△H_1 J/g	△H_2 J/g	T_{max} ℃	Tonset-1 ℃	Tonset-2 ℃	Tonset-3 ℃	Weight Loss-1 %	Weight Loss-2 %	Weight Loss-3 %	Residual %
TPU@None	18.11	1261.89	427.02	227.80	404.80	434.95	36.05	40.77	20.56	2.62
TPU@Rd	167.02	1454.81	414.03	221.69	395.18	436.90	37.04	40.50	19.22	3.24
TPU@Rc	0	2475.14	415.98	225.06	330.07	440.15	19.02	58.67	19.68	2.63
TPU@Rd_Rc	82.87	1457.22	409.87	227.40	399.99	441.97	35.49	40.59	20.25	3.67
TPU@nAg#Rd_Rc	0	1063.32	391.94	288.88	370.88	433.91	27.17	42.24	29.83	0.76

Table S3. The sequences of primers used for quantitative real-time PCR.

Gene	Primer (5′⟶3′)
Actb	F: CATCCGTAAAGACCTCTATGCCAAC
	R: ATGGAGCCACCGATCCACA
Il1b	F: TGCCACCTTTTGACAGTGATG
	R: TGATGTGCTGCTGCGAGAT
Tnfa	F: AAGCCTGTAGCCCACGTCGTA
	R: GGCACCACTAGTTGGTTGTCTTTG
Il6	F: CCACTTCACAAGTCGGAGGCTTA
	R: CCAGTTTGGTAGCATCCATCATTTC
